# Super-dominant pathobiontic bacteria in the nasopharyngeal microbiota as causative agents of secondary bacterial infection in influenza patients

**DOI:** 10.1080/22221751.2020.1737578

**Published:** 2020-03-17

**Authors:** Tian Qin, Taoran Geng, Haijian Zhou, Yang Han, Hongyu Ren, Zhifeng Qiu, Xudong Nie, Tiekuan Du, Junrong Liang, Pengcheng Du, Wei Jiang, Taisheng Li, Jianguo Xu

**Affiliations:** a State Key Laboratory for Infectious Disease Prevention and Control, Collaborative Innovation Centre for Diagnosis and Treatment of Infectious Diseases, National Institute for Communicable Disease Control and Prevention, Chinese Center for Disease Control and Prevention, Beijing, People’s Republic of China; b Shanghai Institute for Emerging and Re-emerging Infectious Diseases, Shanghai Public Health Clinical Centre, Shanghai, People’s Republic of China; c Department of Infectious Diseases, Peking Union Medical College Hospital, Chinese Academy of Medical Sciences and Peking Union Medical College, Beijing, People’s Republic of China; d Beijing Key Laboratory of Emerging Infectious Diseases, Institute of Infectious Diseases, Beijing Ditan Hospital, Capital Medical University, Beijing, People’s Republic of China; e Department of Laboratorial Science and Technology, School of Public Health, Peking University, Beijing, People’s Republic of China; f Research Unit of New Microbes, Chinese Academy of Medical Sciences, Beijing, People’s Republic of China

**Keywords:** Influenza patients, lower-respiratory tract infection, nasopharyngeal microbiota, 16S rRNA V3–V4, super-dominant pathobiontic genus

## Abstract

The source of secondary lower respiratory tract bacterial infections in influenza patients is not fully understood. A case–control study was conducted during the 2017–2018 influenza epidemic period in Beijing, China. Nasopharyngeal swabs were collected from 52 virologically confirmed influenza patients and 24 healthy medical staff. The nasopharyngeal microbiota taxonomic composition was analysed using high-throughput sequencing of the 16S rRNA gene V3–V4 regions. The super-dominant pathobiontic bacterial genus (SDPG) was defined as that accounting for >50% of sequences in a nasopharyngeal swab. We attempted to isolate bacteria of this genus from both nasopharyngeal swabs and lower-respiratory tract samples and analyse their genetic similarities. We observed a significantly lower taxonomy richness in influenza cases compared with healthy controls. A SDPG was detected in 61% of severe cases but in only 24% of mild cases and 29% of healthy controls. In 10 cases, the species isolated from lower-respiratory tract infection sites were identified as belonging to the nasopharyngeal microbiota SDPG. Genetically identical strains were isolated from both nasopharyngeal swabs and lower-respiratory tract infection sites, including 23 *Acinetobacter baumannii* strains from six severe cases, six *Klebsiella pneumoniae* strains from two severe cases, five *Pseudomonas aeruginosa* strains from one severe and one mild case, and four *Corynebacterium striatum* strains from two severe cases. The SDPG in the nasopharyngeal microbiota are the likely cause of subsequent infection in influenza patients.

## Introduction

Influenza is a major cause of epidemic and pandemic respiratory infection, causing considerable disease and a high death toll over a short period of time [1]. Subsequent bacterial pneumonia was responsible for millions of deaths during the influenza pandemics of 1918 [2,3] and 2009 [4]. The upper respiratory tract microbiota is considered a gatekeeper of respiratory health, preventing or resisting the colonization of invading respiratory pathogens [5], including pathobionts [6] such as *Streptococcus pneumoniae* [7], *Haemophilus influenzae* [8], *Neisseria meningitidis* [9], and *Staphylococcus aureus* [10], which can act as harmless commensals or as highly invasive and deadly pathogens depending on the circumstances.

It was recently proposed that respiratory infections are linked to an imbalance of the nasopharyngeal microbiota [11–13]. A study showed that that pneumonia in the older population and young adults was associated with dysbiosis of the upper respiratory tract microbiome with bacterial overgrowth of a single species and the absence of distinct anaerobic bacteria [12]. Another study suggested that nasopharyngeal microbiota dysbiosis was associated with respiratory syncytial virus infection and disease severity in children [13]. However, whether the observed microbiome changes are a cause or a consequence of the development of disease or merely coincide with disease status remains a question for future research.

During the seasonal influenza epidemic in Beijing, China that spanned from December 2017 to March 2018, the number of influenza virus-related deaths was seven times higher than that in the 2016–2017 epidemic (http://ivdc.chinacdc.cn/cnic/) (Figure S1). Many recorded deaths were due to subsequent severe bacterial pneumonia. We hypothesized that the responsible pathogenic bacteria originated from the nasopharyngeal microbiota. Here, we investigate whether a super-dominant pathobiontic genus (SDPG) in the nasopharyngeal microbiota was associated with secondary bacterial infection in influenza virus-infected patients.

## Materials and methods

### Study design

A total of 113 virologically confirmed influenza patients were hospitalized in Peking Union Medical College Hospital, Beijing, China, from December 2017 to March 2018. Among them, the 52 patients who provided at least one nasopharyngeal swab during their hospitalization were included in this study (Table S1). The mean patient age was 56 ± 19 years old, and 54% were male. Nasopharyngeal swabs were also collected from a control group of 24 healthy individuals. The mean age of the control group was 59 ± 17 years old, and 54% were male. The nasopharyngeal microbiota compositions of the swabs were analysed. Comparison and isolation of bacterial pathogens from both the lower-respiratory tract samples and nasopharyngeal swabs were conducted.

The nasopharyngeal samples were collected from every subject using collection swabs and placed in a collection tube for nasopharyngeal swabs (Hope Bio-Technology Co., Ltd., Qingdao, China) containing 3 ml of phosphate-buffered saline (PBS). The tubes containing nasopharyngeal specimens were immediately placed into an icebox and transported to the laboratory where DNA extraction was performed.

### High-throughput sequencing, annotation, and analysis of the 16S rRNA gene

The total genomic DNA from the nasopharyngeal swabs was extracted using the CTAB/SDS method. The 16S rRNA gene V3–V4 region was amplified by polymerase chain reaction (PCR), sequenced, annotated, and analysed as described previously (Supplemental Methods) [14]. The V3–V4 region of the 16S rRNA gene was amplified by PCR using universal primers (F: 5'-CCTAYGGGRBGCASCAG-3’, R: 5'-GGACTACNNGGGTATCTAAT-3’) with a 6-bp barcode unique to each sample. The resulting amplicons were purified, pooled, and sequenced on an Illumina HiSeq 2500 PE-250 platform (Illumina, San Diego, CA, USA) using pair-end sequencing (2* *×* *250 bp).

In order to remove possible contamination, two quality control methods were implemented. The first step is to filter out operational taxonomic units (OTUs) with relative abundance (RA) less than 0.1%. Meanwhile, 16 negative controls, including 10 swabs not sampled, three ddH_2_O and three phosphate buffer saline (PBS) samples, were tested. These blank controlling samples were used to extract bacterial genomic DNA and were sequenced using the same batch of reagents and consumables in the same laboratory and equipment. The possible contamination based on top 10 OTUs of the negative controls were deleted from the sequences of nasopharyngeal specimens.

Alpha- and beta-diversity analyses were performed using QIIME 2 [15]. Principal coordinate analysis (PCoA), non-metric multi-dimensional scaling (NMDS), and permutational analysis of variance (PERMANOVA) analyses were performed using R software (version 2.15.3). For all statistical testing, *P*-values were corrected for multiple testing using the Benjamini and Hochberg method.

### Pathobiontic bacterial species isolation and genetic-relatedness analysis

The isolation and identification of pathobiontic bacteria was attempted from clinical samples, that is, bronchoalveolar lavage fluid (BLF), endotracheal aspirates (ES), sputum, and blood [16]. The genetic relatedness of the resulting isolates was analysed using pulse-field gel electrophoresis (PFGE) [17–22], whole genome sequencing, and sequence comparisons (Supplemental Methods).

### Classification of mild versus severe influenza cases

We divided the influenza virus cases into two subgroups: mild and severe cases. All of the influenza virus infection cases (diagnosed according to the World Health Organization (WHO) guidelines [23]) were classified as mild cases unless they had one or more of the following clinical presentations and thus were classified as severe cases: (1) persistent fever with coughing, expectoration, bloody sputum, or chest pain; (2) tachypnoea, dyspnoea, and cyanosis; (3) somnolence, dysphoria, and convulsion; (4) severe vomiting, diarrhoea, and dehydration; (5) pneumonia; (6) obvious aggravation of underlying diseases; (7) respiratory failure, acute necrotizing encephalopathy, septic shock, or multiple organ dysfunction syndrome [24].

## Results

### Subsequent bacterial infection in >80% of fatal influenza cases

Of the 52 influenza patients who provided nasopharyngeal swabs, 39 (75%) and 13 (25%) were infected by influenza virus A and B, respectively, as confirmed by qRT-PCR detection and sequencing. Thirty-one (60%) patients were classified as severe cases, among which 11 (35%) cases were fatal.

A subsequent bacterial infection was diagnosed in patients who had elevated levels of procalcitonin (PCT) or an isolated pathogenic bacterial species in the blood, alveolar lavage fluid, air intubation, or sputum samples. Such infections were recorded for 71% of severe cases and 10% of mild cases (*P *< 0.001). Additionally, subsequent bacterial infections were detected in 82% of fatal cases and 42% of cases in which the patients survived (*P *= 0.003).

### Lower nasopharyngeal microbiota diversity in influenza virus patients

Nasopharyngeal swabs were available for 31 severe cases, 21 mild cases, and 24 healthy subjects. Additionally, two serial swabs were provided by 17 severe and 7 mild cases. From a total of 100 nasopharyngeal swabs, 7,527,825 high-quality bacterial 16S rRNA sequences were obtained (Table S2), ranging in length from 409–429 bp (mean: 421.7 ± 5.1 bp), and 42,070–98,149 (mean: 71,693) sequences/swabs were obtained. After filtering out OTUs with RA less than 0.1% and six possible contamination taxonomies based on top 10 OTUs of the blank controls (*Enterobacteriaceae*, *Methylobacterium*, *Sphingomonas*, *Faecalibacterium*, *Microbacterium*, *Enterococcus durans*), thee sequences were clustered into 5,174 OTUs with 97% identity, with an average of 197.2 ± 98.8 OTUs/swab (Table S2). The average OTU number for the healthy subjects, mild cases, and severe cases was 293.8 ± 86.8, 197.6 ± 76.3, and 251.7 ± 104.1, respectively.

**Figure 1.**
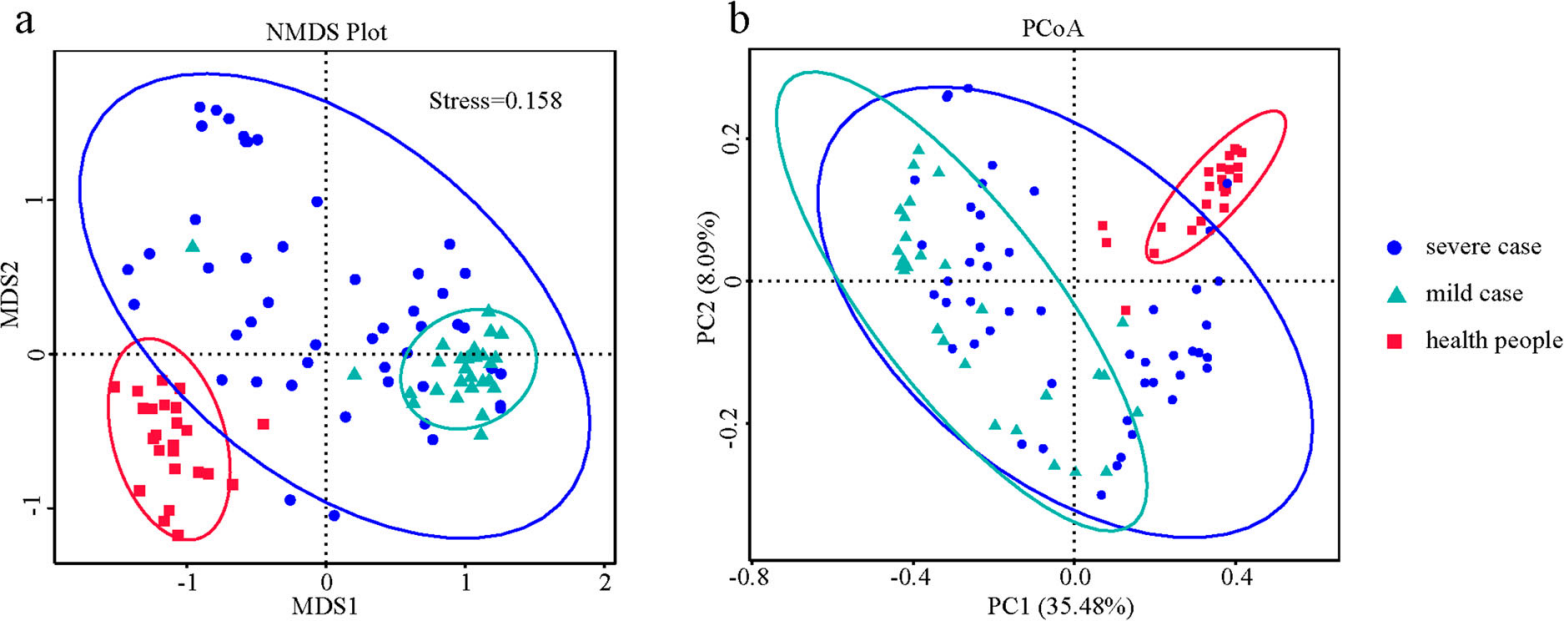
Nasopharyngeal microbiota profiles of severe or mild influenza virus cases and healthy subjects. (a) Non-metric multi-dimensional scaling (NMDS) plot analysis. (b) Principal coordinate analysis (PCoA) plot. PC1: first principal component; PC2: second principal component.

Of the six alpha diversity indices used to analyse the nasopharyngeal microbiota, most indices for the mild and severe case groups were lower than those for the healthy subject group. Specifically, the richness indices (i.e. the number of observed species), Chao1, and Abundance Coverage-based Estimator (ACE) were significantly lower in the mild influenza group (*P *< 0.001) and severe influenza group (*P *< 0.05). The evolutionary distance index (whole-tree phylogenetic diversity) between the influenza virus cases and healthy subjects was also significantly different (*P *< 0.001). However, no significant difference was observed among the three groups for the two evenness indices (Shannon, Simpson) (Figure S2, Table S3). Additionally, there were also significant differences in the richness (Chao1 and ACE) and evolutionary distance indices between the mild and severe case groups. The NMDS and PCoA analyses revealed that the components and structure of the nasopharyngeal microbiota were significantly different between healthy subjects and influenza cases, as well as between mild and severe influenza cases (Figure 1). This finding was further supported by the results of ANOVA (*P *< 0.001) and permutational analysis of variance (PERMANOVA) (*P *< 0.001).

### Dominant pathobiontic genus in the nasopharyngeal microbiota of influenza virus cases

Overall, 26 phyla (7–16 for each sample), 34 classes (11–24 for each samples), 85 orders (22–53 for each sample), 155 families (31–92 for each sample), and 346 genera (49–164 for each sample) were annotated for the 5174 OTUs (122–639 for each sample). The top four most-abundant phyla were Firmicutes, Actinobacteria, Proteobacteria, and Bacteroidetes, together accounting for 96.4%, 86.6%, and 92.6% of the sequences in the healthy control, mild case, and severe case groups, respectively.

**Figure 2.**
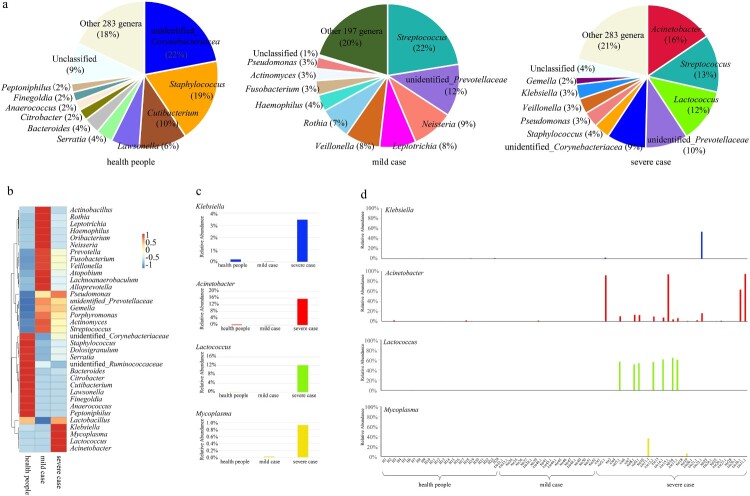
Most-abundant genera in the nasopharyngeal microbiota from severe or mild influenza cases and healthy subjects. (a) The top ten most-abundant genera in the nasopharyngeal microbiota of healthy subjects (left), mild influenza cases (middle), and severe influenza cases (right). (b) Heatmap of the 35 most-abundant genera in the same three groups. (c) Relative abundance of the genera *Klebsiella, Acinetobacter*, and *Lactococcus* in the severe influenza case, mild influenza case, and heathy subject groups. (d) Relative abundance of the genera *Klebsiella*, *Acinetobacter*, and *Lactococcus* in individuals.

The dominant genera, based on RA analysis, differed remarkably among the groups (Figure 2(A)). In the healthy subjects, the top five most-abundant genera were unidentified*_Corynebacteriacea* (22%), *Staphylococcus* (19%), *Cubibacterium* (10%), *Lawsonella* (6%), and *Serratia* (4%). In the mild-case group, the top five most-abundant genera were *Streptococcus* (22%),unidentified*_Prevotellaceae* (12%), *Neisseria* (9%), *Leptotrichia* (8%), and *Veillonella* (8%). For the severe-case group, the top five most-abundant genera were *Acinetobacter* (16%), *Streptococcus* (13%), *Lactococcus* (12%), unidentified*_Prevotellaceae* (10%), and unidentified*_Corynebacteriacea* (9%). Notably, the genera *Prevotella* and *Streptococcus* were dominant in both the mild and severe case groups but not in the healthy control group.

We next compared the RA changes in the top 35 most-dominant genera across the three groups. The RA of four genera (*Klebsiella*, *Acinetobacter*, *Lactococcus* and *Mycoplasma*) were significantly higher in the severe group compared with the healthy control group (*P *< 0.01) (Figure 2(B,C)). We further analysed the RA values of *Klebsiella*, *Acinetobacter*, *Lactococcus* and *Mycoplasma* in each sample. The RA of one, four, seven and one severe group samples was significantly higher than that of the others for *Klebsiella*, *Acinetobacter*, *Lactococcus* and *Mycoplasma*, respectively (*P *< 0.01).

### Super-dominant pathobiontic genus (SDPG) in the nasopharyngeal microbiota

Here, we defined the genus accounting for >50% of the RA in a given individual swab as a SDPG. It was found that 61% of severe cases carried a SDPG, e.g. *Lactococcus* (*n* = 7), *Acinetobacter* (*n* = 4), *Streptococcus* (*n* = 3), unidentified*_Corynebacteriaceae* (*n* = 3), *Staphylococcus* (*n* = 1), or unidentified*_Prevotellaceae* (*n* = 1). By contrast, only 29% and 24% of healthy subjects and mild cases, respectively, carried a SDPG. For the healthy group, the SDPG were unidentified*_Corynebacteriaceae* (*n* = 3), *Staphylococcus* (*n* = 2), *Citrobacter* (*n* = 1), and *Serratia* (*n* = 1). For the mild case group, the SDPG included *Streptococcus* (*n* = 4) and *Pseudomonas* (*n* = 1). The number of cases with a SDPG was statistically different between the severe case and mild case groups (*P *< 0.05) and between the severe case and healthy control groups (*P *< 0.05). However, no such difference was observed between the mild case and healthy control groups (Table S4).

### Some patients showed an identical SDPG across two serial nasopharyngeal swabs

**Figure 3.**
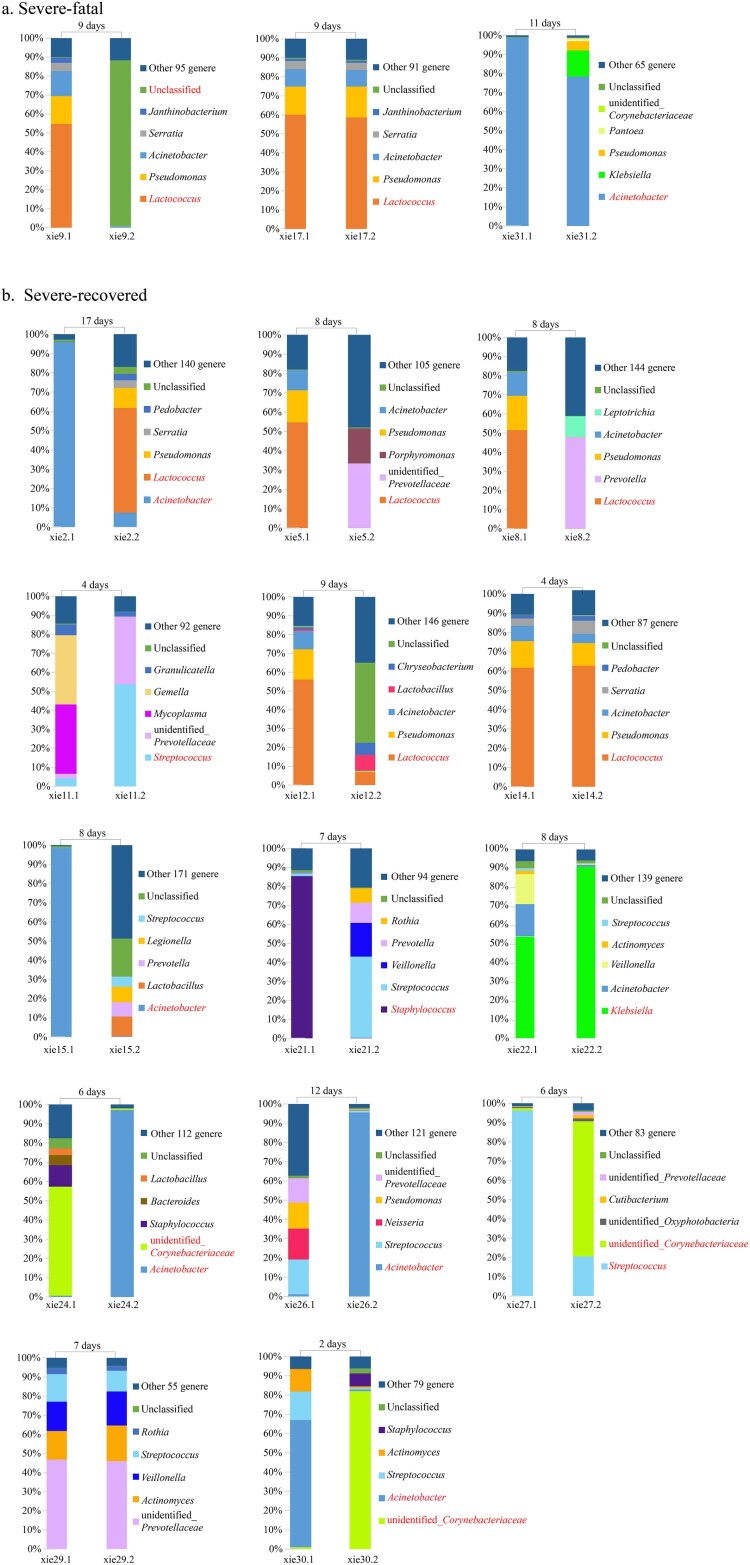
Nasopharynx microbiota of severe influenza cases who provided two nasopharyngeal swabs. Seventeen of the severe influenza cases provided two nasopharyngeal swabs. The top five dominant genera in each nasopharyngeal swab sample were determined. For samples with a SDPG, the genus name is indicated in red text. The length of time specified above the bars indicates the length of time between sample collection. (a) Subgroup of the three patients in the severe case group who died. (b) Subgroup of the 14 patients in the severe group who recovered.

Twenty-four patients (17 severe and 7 mild cases) provided two serial nasopharyngeal swabs during their hospitalization. We studied the possible relationship between disease outcome and the SDPG in the nasopharyngeal microbiota by analysing the 17 available pairs of nasopharyngeal swabs from severe influenza cases. We split the severe cases into subgroups (“severe-recovered” and “severe-fatal” cases) based on their clinical outcome of recovery or death, respectively (Figure 3). A SDPG was detected in all six (100%) nasopharyngeal swabs from the three severe-fatal cases. The SDPG in both swabs from cases #17 and #31 were *Lactococcus* and *Acinetobacter*, respectively (Figure 3(A)). An SDPG was detected in 13 (93%) of the 14 severe-recovered cases who provided two serial swabs (19 of 28 total swabs). In these 13 cases, the SDPG (*Acinetobacter* [*n* = 5], *Lactococcus* [*n* = 5], unidentified*_Corynebacteriaceae* [*n* = 3], *Streptococcus* [*n* = 2], *Staphylococcus* [*n* = 1], and *Klebsiella* [*n* = 1]) was detected in eleven of the first swabs and eight of the second swabs (Figure 3(B)). For case #14, the SDPG in both swabs was *Lactococcus*.

### Pathobiontic bacterial species isolated from both lower-respiratory tract samples and the nasopharyngeal microbiota

**Figure 4.**
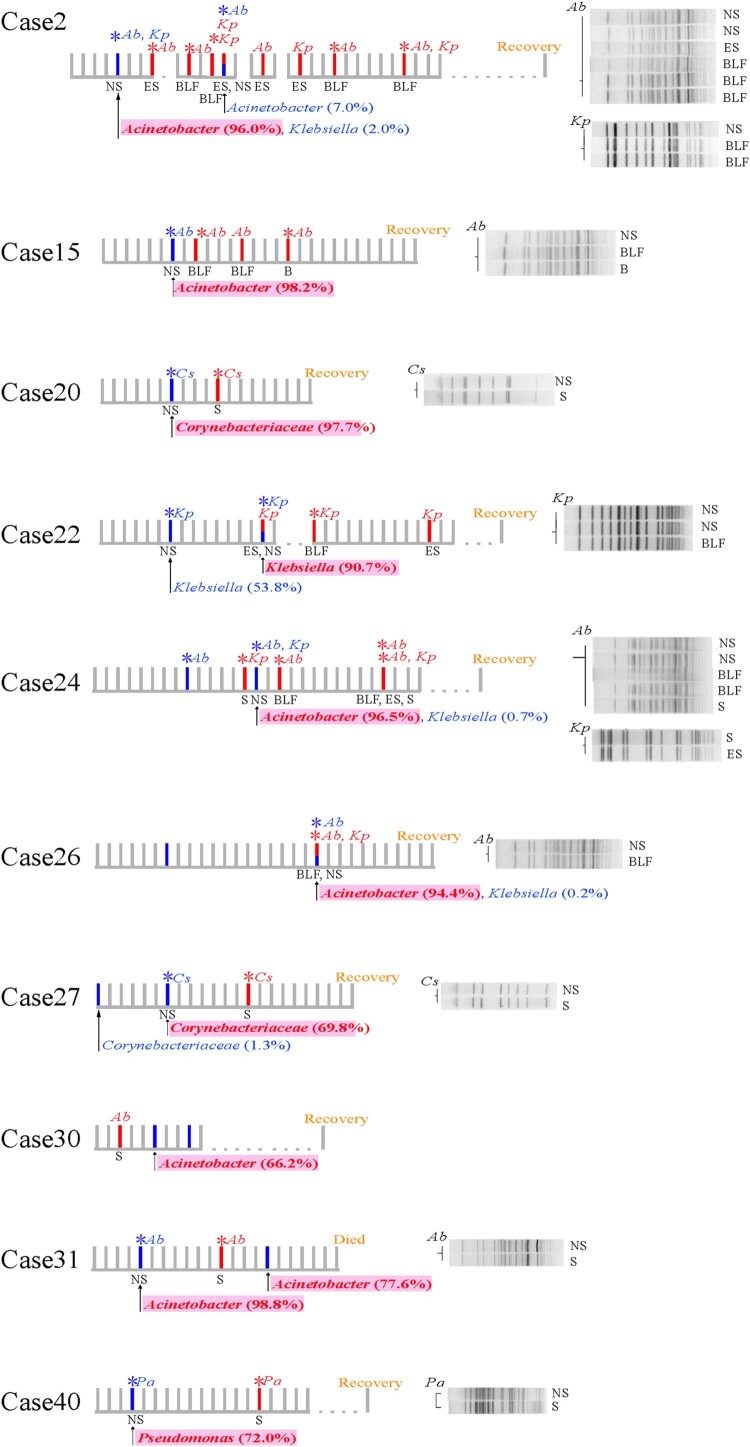
Timeline of cases where the isolated pathobiontic bacterial species belonged to the nasopharyngeal microbiota SDPG. Segments with a solid line represent one day during the disease course; segments with a dotted line represent 5 days during the disease course. Where possible, the PFGE patterns of strains isolated from a given patient are shown to the right of the timeline. SDPG are indicated below each timeline, in text highlighted in light red. Red vertical line indicates the day of clinical strain isolation; blue vertical line indicates the day of nasopharyngeal swab collection; red asterisk indicates the clinical strain analysed by PFGE; blue asterisk indicates the strain isolated from a nasopharyngeal swab and analysed by PFGE. *Ab*, *Acinetobacter baumannii*; *Kp*, *Klebsiella pneumoniae*; *Pa*, *Pseudomonas aeruginosa*; *Cs*, *Corynebacterium striatum*; NS, nasopharyngeal swab; ES, endotracheal aspirates; BLF, bronchoalveolar lavage fluid, S, sputum; B, blood.

Bacterial pathogens were isolated from lower-respiratory tract infection sites for 18 of the 31 severe cases and two of the 21 mild cases, via BLF, ES, sputum, or blood samples (Table S5) (*χ*^2^ **=* *10.496, *P *< 0.001). In 10 cases, the bacterial pathogens isolated from the lower-respiratory tract samples were identified as a member species of the nasopharyngeal microbiota SDPG; these included *Acinetobacter baumannii* (*n* = 6), *Corynebacterium striatum* (*n* = 2), *Klebsiella pneumoniae* (*n* = 1), and *Pseudomonas aeruginosa* (*n* = 1) (Figure 4). Therefore, we hypothesized that the SDPG in the nasopharyngeal microbiota were the causative agents of subsequent lower-respiratory tract infections; if correct, the strains isolated from both the lower-respiratory tract infection sites and nasopharyngeal swabs for the same patient should be genetically identical. Among these 10 cases, bacterial pathogens were also isolated from all patient nasopharyngeal swabs, except for case #30.

**Figure 5.**
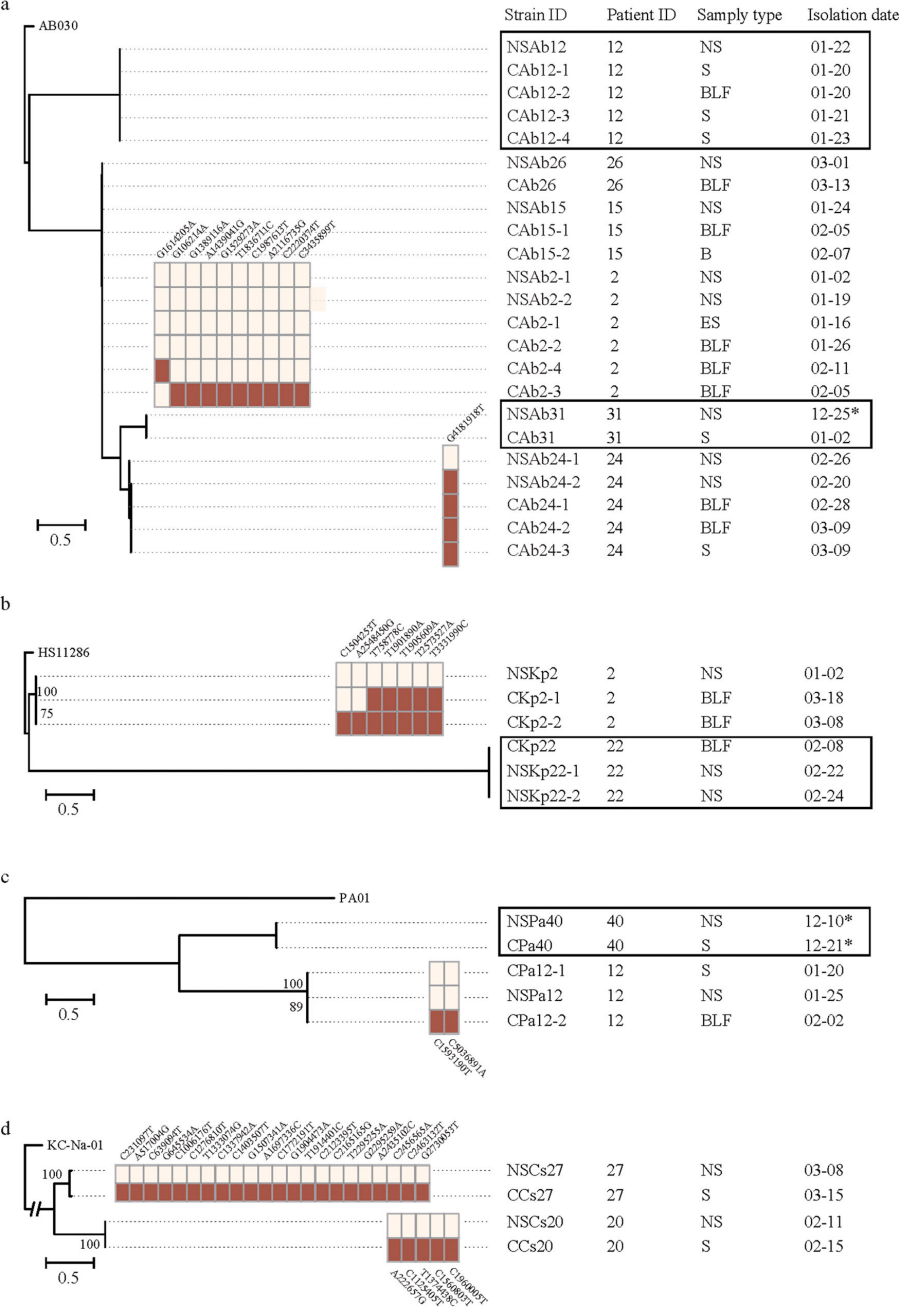
Genome sequences of identical strains isolated from both nasopharyngeal swabs and lower-respiratory tract infection sites. (a–d) Trees based on the genome sequences of the *A. baumannii* (a), *K. pneumoniae* (b), *P. aeruginosa* (c), and *C. striatum* (d) strains isolated from both nasopharyngeal swabs and lower-respiratory tract infection sites. The SNPs between strains from a given patient are shown to the right of the tree. Numbers in the text corresponding to each SNP are the reference genome location. The first base is the base of the first strain of bacteria in each patient, shown in the light-coloured frame. The second base is the mutant base, shown in the dark-coloured frame. Strains with identical sequence are represented by squares. Dates from 2017 are marked with an asterisk; all other dates are from 2018. NS, nasopharyngeal swab; ES, endotracheal aspirates; BLF, bronchoalveolar lavage fluid, S, sputum; B, blood.

For case #2, both *A. baumannii* and *K. pneumoniae* were isolated. The SDPG in the nasopharyngeal swab was *Acinetobacter* (RA: 95.0%). Six isolates with identical PFGE patterns were isolated from different specimens of case #2, including BLF, ES, and nasopharyngeal swabs (Figure 4). Additionally, the whole genome sequences of four of these strains isolated from nasopharyngeal swabs, BLF, and ES were identical (Figure 5(A)). Three *K. pneumoniae* strains with identical PFGE patterns were isolated from a nasopharyngeal swab and BLF (Figure 4). However, whole genome sequence analysis revealed that these three *K. pneumoniae* strains had a few nucleotide variations (Figure 5(B)).

For cases #15, #24, #26, and #31, the SDPG in the nasopharyngeal swabs was *Acinetobacter* (RA > 70%). For case #15, three *A. baumannii* strains with identical PFGE patterns and whole genome sequences were isolated from the BLF, blood, and nasopharyngeal swabs (Figures 4 and 5(A)). For case #24, five *A. baumannii* strains with identical PFGE patterns were isolated from nasopharyngeal swabs, BLF, and sputum (Figure 4), and one nasopharyngeal swab strain had one single nucleotide polymorphism (SNP) (Figure 5(A)). For case #26, *A. baumannii* strains with an identical PFGE pattern and whole genome sequence were isolated from both the BLF and a nasopharyngeal swab (Figures 4 and 5(A)). For case #31, *A. baumannii* strains with an identical PFGE pattern and whole genome sequence were isolated from the sputum and nasopharyngeal swab (Figures 4 and 5(A)).

For case #22, the SDPG in the second swab was *Klebsiella* (RA = 63.4%). Two *K. pneumoniae* strains with an identical PFGE pattern and whole genome sequence were isolated from the BLF and a nasopharyngeal swab (Figures 4 and 5(B)). For case #40, the SDPG in the nasopharyngeal swab was *Pseudomonas* (RA = 70.6%). Two *P. aeruginosa* strains were isolated from a nasopharyngeal swab and sputum, and showed highly similar PFGE patterns and identical whole genome sequences (Figures 4 and 5(C)).

The SDPG in the nasopharyngeal swabs from cases #20 and #27 was *Corynebacterium* (RA = 96.4% and 69.2%, respectively). *C. striatum* strains were isolated from the sputum and a nasopharyngeal swab for both cases #20 and #27. They showed identical PFGE patterns (Figure 4), but a slight nucleotide variation at the genome level was observed (Figure 5(D)).

For the other 10 cases, the pathobiontic species isolated from lower-respiratory tract and/or blood samples was not a member of the nasopharyngeal microbiota SDPG. Only seven of these cases had a SDPG: *Lactococcus* (5 cases), *Streptococcus* (1 case), and *Corynebacterium* (1 case). In cases #11 and #16, *C. albicans* was isolated from the sputum and ES. In four cases, *S. pyogenes* (case #6), *K. pneumoniae* (case #8), *S. epidermidis* (case #17), and *S. haemolyticus* (case #37) were isolated from blood only (Figure S3).

For case #12, the SDPG in the nasopharyngeal swab was *Lactococcus* (RA = 54.0%); however, *Acinetobacter* (RA = 9.8%) and *Pseudomonas* (RA = 15.7%) were also detected*.* Five *A. baumannii* strains showing identical PFGE patterns and genome sequences were isolated from the nasopharyngeal swab, sputum, and BLF (Figure S3, Figure 5(A)). Three *P. aeruginosa* strains were isolated from the nasopharyngeal swab, sputum, and BLF, which showed identical PFGE patterns (Figure S3) and two SNPs were observed in the genome sequence (Figure 5(C)).

Together, four species were isolated from both lower-respiratory tract samples and nasopharyngeal swabs*.* Twenty-six, nine, five, and four strains of *A. baumannii*, *K. pneumoniae*, *P. aeruginosa*, and *C. striatum*, respectively, were isolated from nine, four, two, and two cases, respectively. Strains from a given patient showed identical PFGE patterns that clustered together. The PFGE patterns of strains from different patients clustered separately (Figure S4).

At the genome level, no or only a few SNPs were found between isolates from different types of specimen (Figure 5, Table S6). All *A. baumannii* strains isolated from both lower-respiratory tract infection sites and nasopharyngeal swabs shared identical genome sequences. A single nucleotide variation was observed for one strain of case #24. One of the two nasopharyngeal strains for case #2 had nine SNPs (Figure 5(A)). For *K. pneumoniae*, strains with identical genome sequences were isolated from lower-respiratory tract infection sites and nasopharyngeal swabs for case #22, but not case #2; the strains isolated from infected sites and nasopharyngeal swabs for case #2 had seven SNPs (Figure 5(B)). For *P. aeruginosa*, strains with slightly different genome sequences were isolated from lower-respiratory tract infection sites and nasopharyngeal swabs for case #12 (Figure 5(C)). For *C. striatum*, PFGE identical strains were isolated from cases #27 and #20, but for these cases, 22 and five SNPs were observed, respectively (Figure 5(D)).

## Discussion

The bacterial genera most frequently detected in the nasopharyngeal microbiota of healthy adults were *Corynebacterium*, *Dolosigranulum*, *Staphylococcus*, and *Streptococcus* [25], which include the well-recognized respiratory pathogens *S. aureus* [26], *S. pneumoniae* [27], *Dolosigranulum pigrum* [28], and *Corynebacterium propinquum/pseudodiphtheriticum* [29]. Notably, the human nasopharyngeal microbiota is composed of both true commensal bacteria and pathobiontic species, which can act as harmless colonizing microbes or as highly invasive pathogens depending on environmental circumstances [30,31]. We report here that bacterial species belonging to the SDPG emerged from the nasopharyngeal microbiota of influenza virus-infected patients, subsequently causing severe pneumonia and bacteraemia with high mortality [32].

Our data indicate that the nasopharyngeal microbiota diversity was significantly lower in influenza virus patients than in healthy control subjects. The OTU numbers and alpha diversity indices for the influenza groups were lower than those for the healthy control group. We previously hypothesized that over-rich pathobiontic bacteria may change their status from co-existence with the host to the promotion of clinical disease [25]. Here, we found that 61% of severe influenza cases had a SDPG in their nasopharyngeal microbiota. Furthermore, by studying the possible relationship between disease outcome and the SDPG in the nasopharyngeal microbiota by analysing the available pairs of nasopharyngeal swabs from severe influenza cases, we found that the continuous existence of a SDPG played a role in the second fatal bacterial infection that followed influenza disease.

To test this possibility, we attempted to isolate the pathobiontic bacterial species from clinical samples and nasopharyngeal swabs, and then compared their genetic relatedness. A finding that the pathogens isolated from the infection sites were genetically identical to those isolated from the nasopharyngeal swabs would support the idea that the nasopharyngeal microbiota SDPG was responsible for the secondary bacterial infection. Four species were isolated from both the lower-respiratory tract samples and nasopharyngeal swabs: *A. baumannii*, *K. pneumoniae*, *P. aeruginosa*, and *C. striatum.* Among the cases in which pathobionts were isolated from the infected sites but not from the nasopharyngeal swabs, most of the pathobionts were fungal species or were isolated only from blood, suggesting that they originated from outside of the nasopharynx.

Contamination with the nasopharyngeal microbiota is a well-known issue in collecting lower respiratory tract samples, such as sputum and BLF. It should therefore be acknowledged that cases where the SDPG in the nasopharynx was also found in the lower respiratory tract could be attributed to contamination. However, in this study, only in a small proportion of cases was the SDPG in the nasopharynx also isolated from the sputum and BLF samples. Furthermore, *Lactococcus* was the SDPG in seven cases but no *Lactococcus* was isolated from the lower respiratory tract of these cases. These results showed that there were no problems with our sampling method and that the pathogenic bacteria found in the lower respiratory tract samples were not contaminants.

One limitation of this study was that we were unable to trace whether these SDPG, especially the pathogenic strains, were due to nosocomial infection or an increase in existing colonizing strains. Because the background nasopharyngeal microbiota of these influenza patients before hospitalization was unknown, it was difficult to address this issue. Despite this, our results confirmed that some of the pathogens that cause pneumonia and bacteraemia originated from the nasopharynx.

In summary, for 10 cases of influenza virus infection, pathobiontic bacterial strains with identical PFGE patterns and highly similar whole genomic sequences were isolated from both the infected site and from nasopharyngeal swabs, and among these strains, nine were members of the nasopharyngeal microbiota SDPG (Figure S5). These data are the first evidence that a pathobiontic bacterial species in the nasopharyngeal microbiota (*A. baumannii*, *K. pneumoniae*, *P. aeruginosa*, or *C. striatum*) may opportunistically cause severe secondary infection in patients with influenza*.* The results of this study suggest that these pathogens should be screened and eliminated from the upper respiratory tract to prevent secondary bacterial infection, especially when these bacteria become the SDPG of the nasopharyngeal microbiota.

## Supplementary Material

Supplemental Material

## Data Availability

The data generated in this Whole Genome Shotgun project have been deposited in the NCBI BioProject repository [accession number PRJNA544998] and the BioSample database [accession numbers SAMN11811853–SAMN11811890].
