# Supplementary material for: Super-dominant pathobiontic bacteria in the nasopharyngeal microbiota as causative agents of secondary bacterial infection in influenza patients
Source: Emerg Microbes Infect. 2020 Mar 17;9(1):605–15. doi: 10.1080/22221751.2020.1737578 (PMC7144213; doi:10.1080/22221751.2020.1737578)
Supplement: Supplemental Material [file TEMI_A_1737578_SM4647.docx]

**Supplement**

Supplemental Methods ------------------------------------------------------------------2

Figure S1-----------------------------------------------------------------------------------7

Figure S2-----------------------------------------------------------------------------------7

Figure S3-----------------------------------------------------------------------------------8

Figure S4-----------------------------------------------------------------------------------9

FigureS 5-----------------------------------------------------------------------------------10

Table S1-----------------------------------------------------------------------------------11

Table S2-----------------------------------------------------------------------------------14

Table S3-----------------------------------------------------------------------------------19

Table S4-----------------------------------------------------------------------------------20

Table S5-----------------------------------------------------------------------------------22

Table S6-----------------------------------------------------------------------------------29

**Supplemental Methods**

**Classification of severe and mild influenza cases**

All influenza infection cases were diagnosed in accordance with the WHO guidelines^1^. Unless the case fit the definition of a severe influenza case, as described in “Diagnostic and treatment protocol for influenza (2018 version)” laid down by National Health and Family Planning Commission of the People’s Republic of China^2^, it was categorized as a mild case. Cases with one or more of the following clinical presentations were classified as severe cases: (1) persistent fever over 3 days, accompanied by coughing, expectoration, bloody sputum, or chest pain; (2) tachypnea, dyspnea, and cyanosis; (3) altered mental status: somnolence, dysphoria, and convulsion; (4) severe vomiting, diarrhea, and dehydration; (5) pneumonia; (6) aggravation of underlying disease; and (7) any one of the following presentations: respiratory failure, acute necrotizing encephalopathy, septic shock, or multiple organ dysfunction syndrome.

**High-throughput sequencing, annotation, and analysis of the 16S rRNA gene**

The nasopharyngeal swabs were sampled into tubes containing 3 ml of phosphate-buffered saline, which were immediately placed into an icebox and subsequently frozen at −70 °C in a freezer until use. The total genomic DNA was extracted from the nasopharyngeal swabs using CTAB/SDS method. DNA concentration and purity was monitored on 1% agarose gels. According to the concentration, DNA was diluted to 1ng/μL using sterile water. The V3–V4 region of the 16S rRNA gene was amplified by PCR using universal primers (F: 5'-CCTAYGGGRBGCASCAG-3', R: 5'-GGACTACNNGGGTATCTAAT-3') with a 6-bp barcode unique to each sample. The single amplifications were performed in 25 µl reactions with 50 ng of template DNA. The resulting amplicons were purified, pooled, and sequenced on an Illumina HiSeq 2500 PE-250 platform (Illumina, San Diego, CA, USA) using pair-end sequencing (2 × 250 bp). Barcodes and sequencing primers were trimmed before the paired end reads were merged using FLASH (V1.2.7, http://ccb.jhu.edu/software/FLASH/). Quality filtering was performed using QIIME^3^. Chimeric and incomplete extension sequences generated in the PCR process were filtered out using the UCHIME algorithm (UCHIME Algorithm, http://www.drive5.com/usearch/manual/uchime_algo.html). We set up a negative control with water in the DNA extraction step. However, there were no fragments in the 16S amplification. So we have not sequence the negative control sample.

The 16s rDNA sequences with ≥97% similarity were clustered into operational taxonomic units (OTUs) by using Uparse software (Uparse v7.0.1001, <http://drive5.com/uparse/>). Representative sequences for each OTU were screened for further annotation using the RDP-classifier (Version 2.2, <http://sourceforge.net/projects/rdp-classifier/>) against the SILVA_123 database with an 80% confidence level. OTU representative sequences were identified using MUSCLE software (Version 3.8.31), and a phylogenetic tree was built using the FastTree algorithm. The sequences were then analyzed using the Uparse software (Uparse v7.0.1001, <http://drive5.com/uparse/>).

Alpha- and beta-diversity analyses were performed using QIIME^3^, and the results were displayed using R software. Principal coordinate analysis (PCoA), non-metric multi-dimensional scaling (NMDS), and permutational analysis of variance (PERMANOVA) analyses were performed in R software (Version 2.15.3). For all statistical testing of 16S rRNA data, *P*-values were corrected for multiple testing using the Benjamini and Hochberg method.

**Pathobiontic bacterial species isolation and genetic relatedness analysis**

The isolation of pathobiontic or pathogenic bacteria was attempted for the available clinical samples, which included blood, bronchoalveolar lavage fluid (BLF), endotracheal aspirates (ES), sputum, and nasopharyngeal swabs^4^. The BAL samples were taken by inserting a protected double-lumen catheter through the oral tube or tracheostoma to a wedge position in the bronchial tree, instilling 60 mL of saline, and then immediately withdrawing the fluid. The ES samples were collected via the endotracheal tube’s inline suction catheter. After flushing the suction catheter with 5 mL of sterile saline, the catheter was advanced into the distal trachea and 5 mL of sterile saline was flushed into the trachea and suctioned back into a Lukens trap.

The taxonomic identification of isolates was performed using a VITEK 2 COMPACT Automatic Microbial Identification System. The final identification was confirmed based on a full-length 16s rDNA sequence analysis. The genetic relatedness of isolates was analyzed with pulse-field gel electrophoresis (PFGE). The restriction enzymes used to digest chromosomal DNA for *Acinetobacter baumannii*, *Klebsiella pneumoniae*, *Pseudomonas aeruginosa*, and *Corynebacterium striatum* were *Apa*I, *Xba*I, *Spe*I, and *Swa*I, respectively^5-8^. The restriction fragments were separated using a CHEF-DRIII system (Bio-Rad Laboratories, CA, USA). The digital images were captured and storied electronically as TIFF files, in specific dataset for various bacterial species. All digital images for specific bacterial species were analyzed separately with BioNumerics version 7.1 (Applied Maths, Kortrijk, Belgium). A similarity analysis of the PFGE patterns was performed by calculating the Dice coefficients (SD)^9^ and clustering was performed using the unweighted-pair group method with average linkages (UPGMA). Clones were defined according to a similarity (Dice) coefficient.

Bacterial strains were sequenced using Illumina sequencing by constructing two paired-end (PE) libraries with average insertion lengths of 500 bp and 2000 bp, respectively. Sequences were generated using an Illumina GA IIx (Illumina Inc., San Diego, CA, USA). Raw data was processed in four steps, including removing reads with 5 bp of ambiguous bases, removing reads with 20 bp of low quality (≤ Q20) bases, removing adapter contamination, and removing duplicated reads. Finally, 100× libraries were obtained with clean PE read data. The single-nucleotide polymorphisms (SNPs) were examined through pairwise comparisons using SOAPsnp^10^. Reads with low quality (>3 consecutive bases with a quality score of ≤Q20) were removed before SNP calling. SNPs were called if they met the following criteria using SOAPsnp45: (i) each SNP site was covered by ≥20 reads, (ii) the distance between two SNP sites was ≥5 bp, (iii) the SNP was not located in a repeat region, and (iv) the prior probability of heterozygous SNPs is ≤0.1%.

**Nucleotide sequence accession number**

This Whole Genome Shotgun project has been deposited into GenBank under the Bioproject ID PRJNA544998, accession numbers SAMN11811853–SAMN11811890.

**Statistical analyses**

The rates of subsequent bacterial infection between the severe cases and mild cases and between the survived and fatal cases were compared using the χ^2^ test with a row × column table (McNemar’s test). The difference in bacterial numbers was analyzed with the non-parametric test of independent samples, using SPSS 11.5 software. Differences were considered significant if they had *P* values of <0.01.

**References for supplemental methods**

1. World Health Organization (WHO). Clinical features of severe cases of pandemic influenza—Pandemic (H1N1) 2009 briefing note 13. https://www.who.int/csr/disease/swineflu/notes/h1n1_clinical_features_20091016/en/index.html. Accessed October 19, 2009.

2. National Health and Family Planning Commission of the People’s Republic of China. Diagnostic and treatment protocol for influenza (2018 version). *Chin J Clin Infect Dis*. 2018;11(1):1-5.

3. Caporaso JG, Kuczynski J, Stombaugh J, et al. QIIME allows analysis of high-throughput community sequencing data. *Nat Methods*. 2010;7(5):335–336.

4. Lagier JC, Edouard S, Pagnier I, Mediannikov O, Drancourt M, Raoult D. Current and past strategies for bacterial culture in clinical microbiology. *Clin Microbiol Rev*. 2015;28(1):208-236.

5. Hammerum AM, Hansen F, Skov MN, et al. Investigation of a possible outbreak of carbapenem-resistant *Acinetobacter baumannii* in Odense, Denmark using PFGE, MLST and whole-genome-based SNPs. J Antimicrob Chemother. 2015;70:1965–1968.

6. Han H, Zhou H, Li H, et al. Optimization of pulse-field gel electrophoresis for subtyping of *Klebsiella pneumoniae*. Int J Environ Res Public Health. 2013;10:2720–2731.

7. Selim S, El Kholy I, Hagagy N, et al. Rapid identification of *Pseudomonas aeruginosa* by pulsed-field gel electrophoresis. Biotechnol Biotechnol Equip. 2015;29:152–156.

8. Wang J, Wang Y, Du X, et al. Rapid transmission of multidrug-resistant *Corynebacterium striatum* among susceptible patients in a tertiary hospital in China. J Infect Dev Ctries. 2016;10:1299–1305.

9. Dice LR. Measures of the amount of ecologic association between species. Ecology. 1945; 26: 297–302.

10. Li R, Li Y, Fang X, et al. SNP detection for massively parallel whole-genome resequencing. *Genome Res*. 2009;19(6):1124-1132.

**Supplemental Figures**


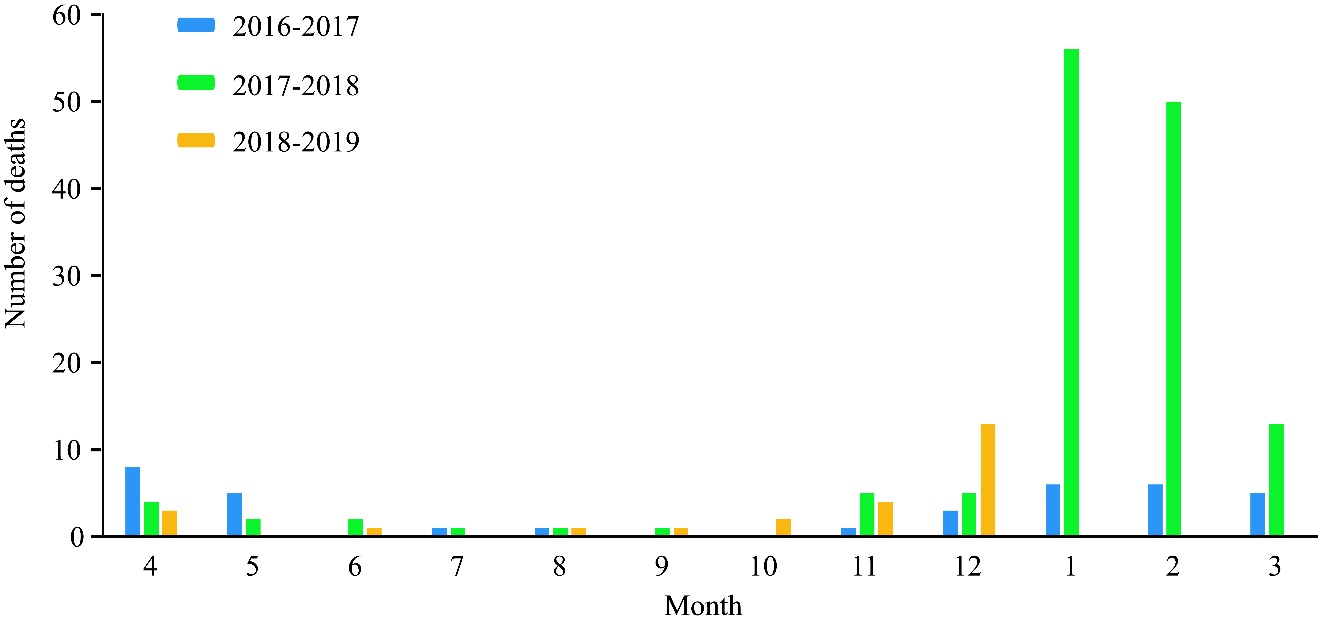


**Figure S1. The number of reported deaths of influenza virus patients in China from 2016–2018**


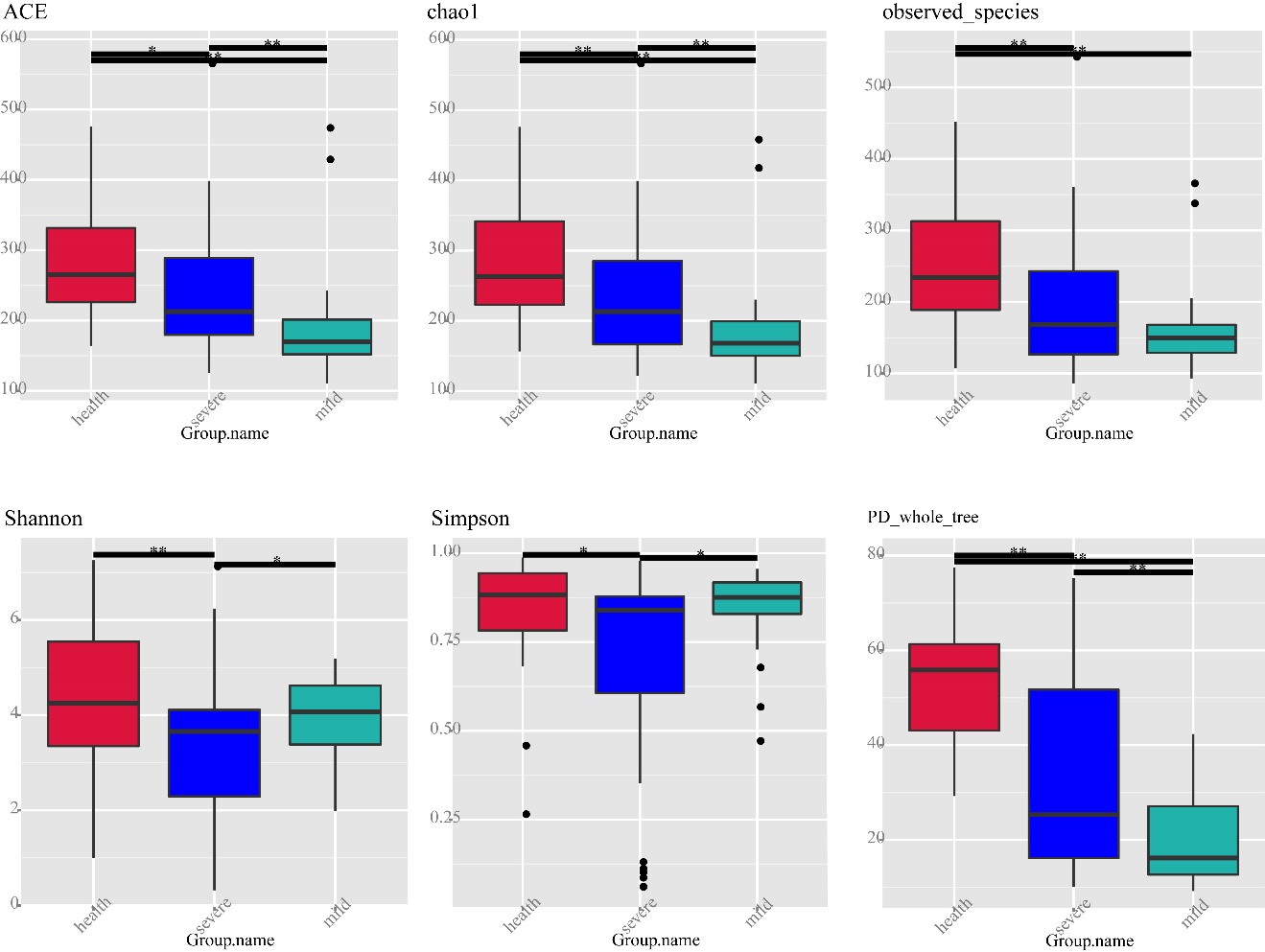


**Figure S2. Alpha diversity analysis of the nasopharyngeal microbiota from the healthy subject, severe-case, and mild-case groups**

**
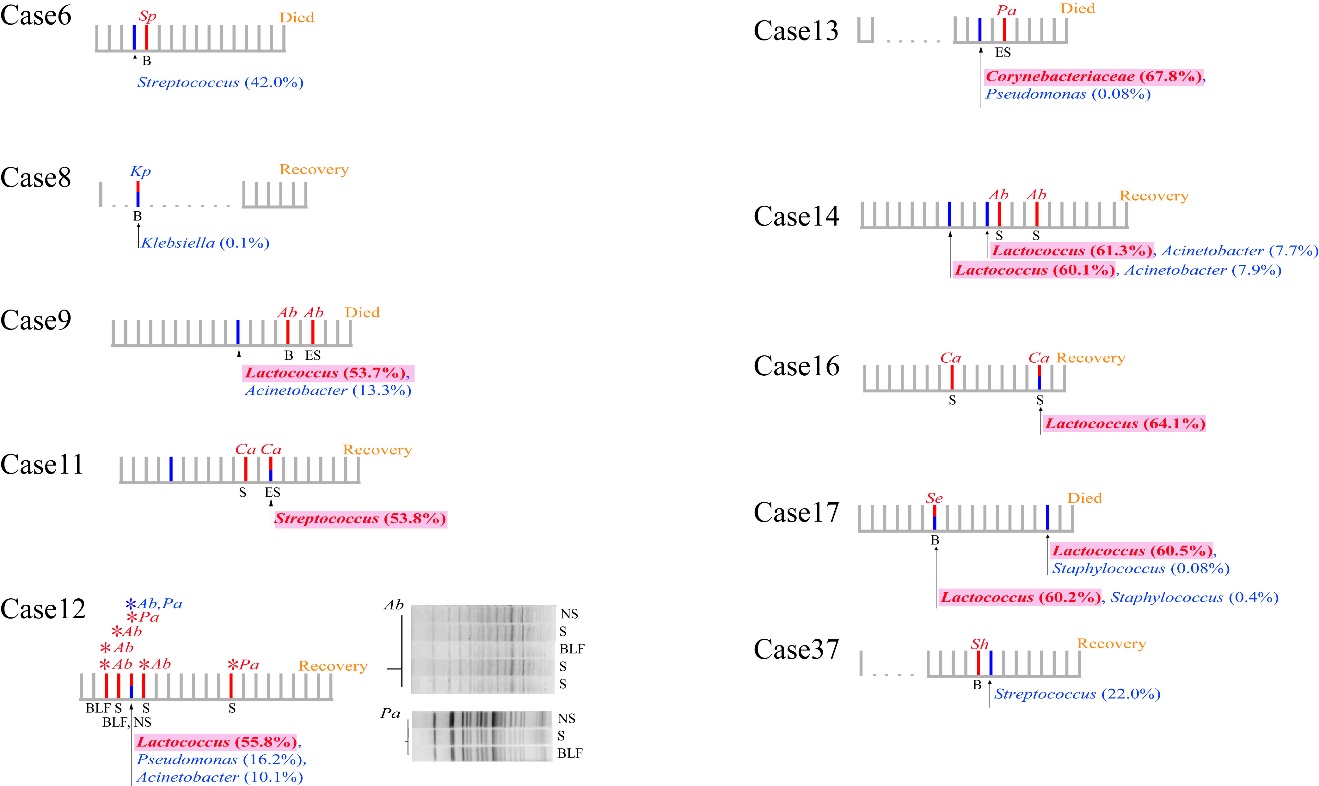
**

**Figure S3. Timeline of cases where the isolated pathobiontic species inconsistency with the nasopharyngeal microbiota SDPG**

Segments with a solid line represent one day during the disease course; segments with a dotted line represent five days during the disease course. Where possible, the PFGE patterns of strains isolated from a given patient are shown to the right of the timeline. SDPGs are indicated below each timeline, in text highlighted in light red. Red vertical line, day clinical strain isolation; blue vertical line, day of nasopharyngeal swab collection; red asterisk, clinical strain analyzed by PFGE; blue asterisk, strain isolated from a nasopharyngeal swab and analyzed by PFGE. *Ab*, *Acinetobacter baumannii*; *Kp*, *Klebsiella pneumoniae*; *Pa*, *Pseudomonas aeruginosa*; *Sp*, *Streptococcus pyogenes*; *Ca*, *Candida albicans*; *Se*, *Staphylococcus epidermidis*; *Sh*, *Streptococcus haemolyticus*; NS, nasopharyngeal swab; ES, endotracheal aspirates; BLF, bronchoalveolar lavage fluid, S, sputum; B, blood.


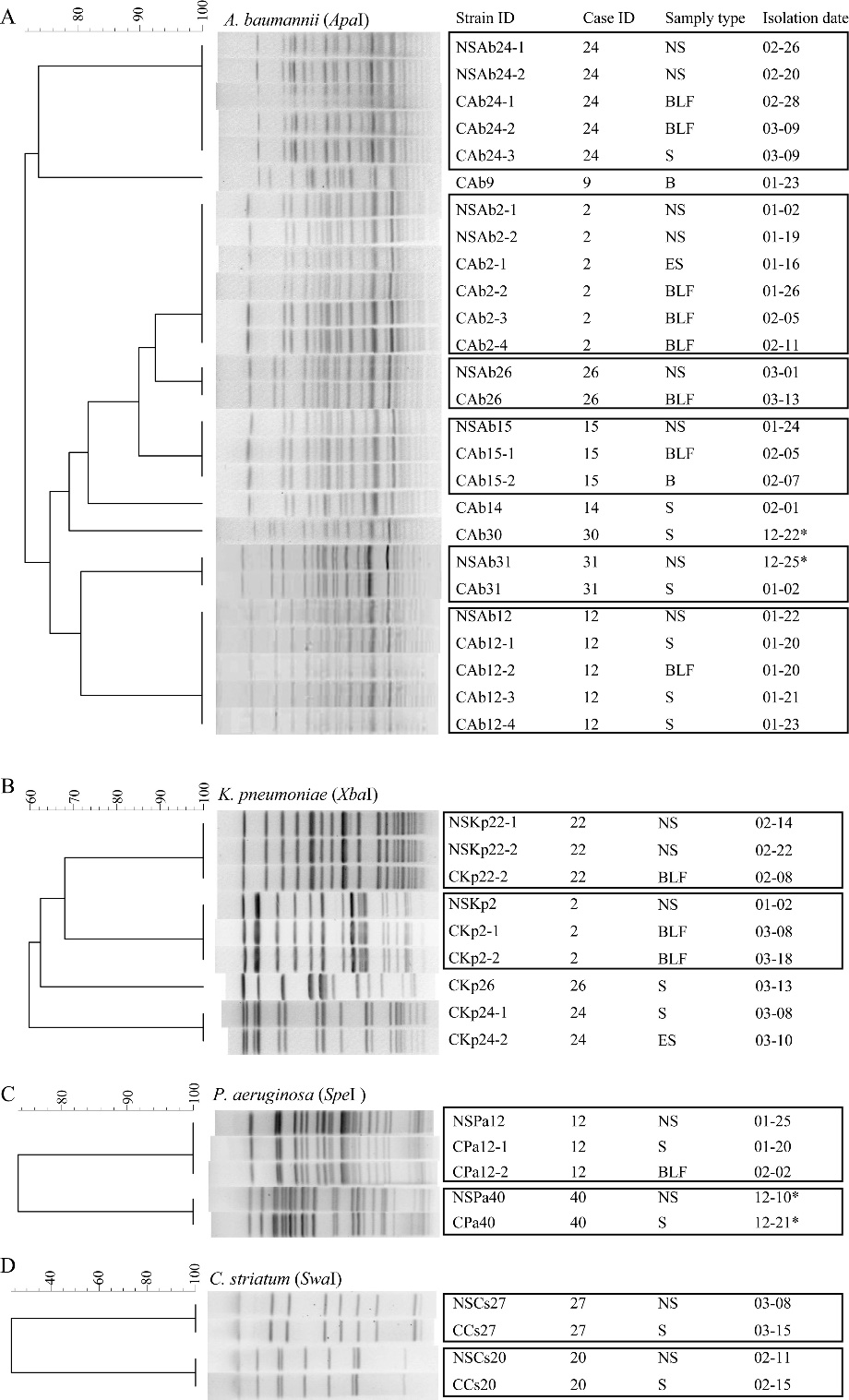


**Figure S4. PFGE patterns of pathobiontic bacterial species isolated from low-respiratory tract and nasopharyngeal swab samples.** Terms in parenthesis after the species name (*ApaI*, *XbaI*, *SpeI*, and *SwaI*) were restriction endonuclease used in PFGE experiments.

(A–D) The PFGE patterns of *Acinetobacter baumannii* (A), *Klebsiella pneumoniae* (B), *Pseudomonas aeruginosa* (C), and *Corynebacterium striatum* (D) strains isolated from nasopharyngeal swabs and low-respiratory tract samples. NS, nasopharyngeal swab; ES, endotracheal aspirates; BLF, bronchoalveolar lavage fluid, S, sputum; B, blood. Dates marked with asterisks (*) are from 2017; all unmarked dates are from 2018.


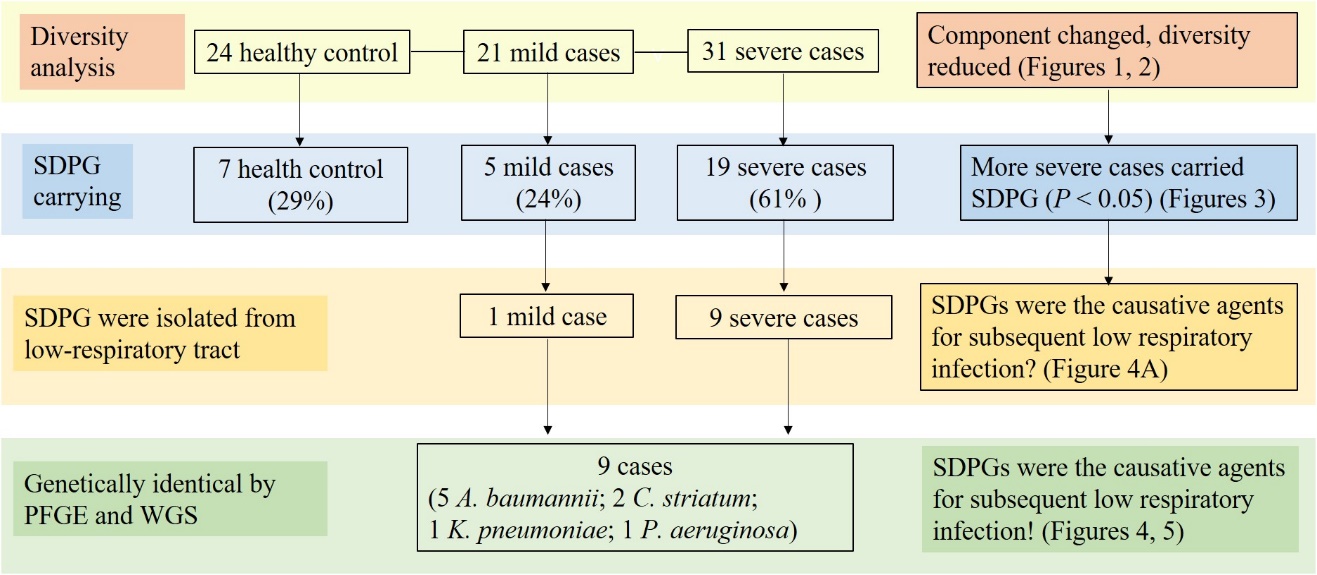


**Figure S5. Schematic summary of the analysis process and major results of this study**

SDPG, Super dominant pathobiontic genus; PFGE, pulse-field gel electrophoresis; WGS, whole genome sequencing.

**Supplemental Tables**

**Table S1. Clinical information of influenza patients in this study**

| **Patient ID** | **Sex** | **Age (years)** | **Severity^a^** | **Influenza virus** | **Outcome** | **Onset of fever** | **First swab sampled** | **Second swab sampled** |
| --- | --- | --- | --- | --- | --- | --- | --- | --- |
| 1 | M | 45 | S | HA | Deceased | 11-30**^b^ | 12-06** |  |
| 2 | F | 35 | S | HB |  | 12-28** | 01-02 | 01-19 |
| 3 | F | 24 | S | HA | Deceased | 12-28** | 01-02 |  |
| 4 | F | 29 | S | HA |  | 01-07 | 01-09 |  |
| 5 | F | 29 | S | HA |  | 01-01 | 01-10 | 01-18 |
| 6 | M | 45 | S | HA | Deceased | 01-10 | 01-13 |  |
| 7 | F | 60 | S | HA |  | 01-04 | 01-13 |  |
| 8 | M | 66 | S | HA |  | 01-14 | 01-15 | 01-23 |
| 9 | M | 64 | S | HA | Deceased | 01-05 | 01-15 | 01-24 |
| 10 | M | 66 | S | HA | Deceased | 01-09 | 01-15 |  |
| 11 | M | 82 | S | HB |  | 01-12 | 01-16 | 01-20 |
| 12 | M | 83 | S | HA |  | 01-18 | 01-25 |  |
| 13 | M | 44 | S | HB | Deceased | 01-22 | 01-27 |  |
| 14 | F | 84 | S | HA |  | 01-21 | 01-28 | 01-31 |
| 15 | F | 64 | S | HB |  | 01-24 | 01-30 | 02-07 |
| 16 | F | 77 | S | HA |  | 01-24 | 02-01 |  |
| 17 | F | 77 | S | HA | Deceased | 01-27 | 01-31 | 02-09 |
| 18 | M | 81 | S | HA | Deceased | 01-15 | 01-31 |  |
| 19 | M | 104 | S | HA | Deceased | 02-01 | 02-07 |  |
| 20 | M | 71 | S | HA |  | 02-05 | 02-11 |  |
| 21 | F | 55 | S | HA |  | 02-07 | 02-13 | 02-20 |
| 22 | M | 78 | S | HB |  | 02-08 | 02-14 | 02-22 |
| 23 | F | 67 | S | HA |  | 02-12 | 02-20 |  |
| 24 | M | 41 | S | HA |  | 02-12 | 02-20 | 02-26 |
| 25 | M | 63 | S | HA | Deceased | 02-20 | 02-28 |  |
| 26 | M | 62 | S | HA |  | 02-22 | 03-01 | 03-13 |
| 27 | F | 56 | S | HB |  | 03-02 | 03-02 | 03-08 |
| 28 | F | 85 | S | HA |  | 03-04 | 03-08 |  |
| 29 | F | 68 | S | HB |  | 03-04 | 03-08 | 03-15 |
| 30 | M | 54 | S | HB |  | 12-20** | 12-26** | 12-28** |
| 31 | M | 54 | S | HB | Deceased | 12-25** | 12-29 | 01-09 |
| 32 | M | 67 | M | HB |  | 01-23 | 01-23 | 01-29 |
| 33 | F | 59 | M | HB |  | 01-18 | 01-23 | 01-25 |
| 34 | F | 58 | M | HA |  | 08-06** | 08-07** |  |
| 35 | M | 46 | M | HA |  | 08-06** | 08-07** |  |
| 36 | M | 77 | M | HA |  | 08-06** | 08-07** |  |
| 37 | F | 57 | M | HA |  | 08-05** | 08-07** |  |
| 38 | F | 36 | M | HA |  | 08-06** | 08-07** | 08-12** |
| 39 | M | 65 | M | HA |  | 08-07** | 08-08** |  |
| 40 | M | 59 | M | HA |  | 12-07** | 12-10** | 12-25** |
| 41 | F | 20 | M | HA |  | 12-10** | 12-10** |  |
| 42 | M | 65 | M | HA |  | 12-10** | 12-10** |  |
| 43 | F | 64 | M | HA |  | 12-10** | 12-10** | 12-18** |
| 44 | M | 70 | M | HA |  | 12-10** | 12-10** | 12-18** |
| 45 | M | 15 | M | HA |  | 12-10** | 12-10** |  |
| 46 | M | 73 | M | HA |  | 12-10** | 12-10** |  |
| 47 | M | 58 | M | HA |  | 12-10** | 12-10** |  |
| 48 | M | 65 | M | HA |  | 12-10** | 12-10** | 12-25** |
| 49 | F | 45 | M | HA |  | 12-09** | 12-10** |  |
| 50 | F | 62 | M | HA |  | 12-10** | 12-10** |  |
| 51 | F | 49 | M | HB |  | 01-22 | 01-23 |  |
| 52 | F | 74 | M | HB |  | 01-28 | 02-01 |  |

^a^ S: severe case group, M: mild case group.

^b^ Dates marked with ** are from 2017 (all unmarked dates are from 2018).

**Table S2. Summary of analyzed sequence information**

| **Sample^a^** | **Q20** | **GC%** | **Raw reads** | **Clean reads** | **Effective%** | **Base (nt)** | **Total tag** | **Average length (nt)** | **Tax tag** | **Unclassified tag** | **Unique tag** | **OTU number** |
| --- | --- | --- | --- | --- | --- | --- | --- | --- | --- | --- | --- | --- |
| H1 | 85.42 | 52.61 | 94928 | 85929 | 90.52 | 36012400 | 79110 | 419 | 66272 | 64 | 12774 | 348 |
| H2 | 84.47 | 54.72 | 57409 | 54130 | 94.29 | 22458102 | 52784 | 414 | 44721 | 4 | 8059 | 226 |
| H3 | 87.11 | 51.23 | 85751 | 80081 | 93.39 | 33723474 | 79140 | 421 | 71787 | 0 | 7353 | 297 |
| H4 | 87.88 | 51.84 | 83811 | 80329 | 95.85 | 33276935 | 79446 | 414 | 71901 | 12 | 7533 | 214 |
| H5 | 84.76 | 52.37 | 50528 | 44050 | 87.18 | 18334771 | 38423 | 416 | 28581 | 200 | 9642 | 464 |
| H6 | 84.09 | 55.68 | 84012 | 80225 | 95.49 | 33817407 | 71883 | 421 | 62671 | 9 | 9203 | 169 |
| H7 | 87.4 | 53.67 | 86021 | 80202 | 93.24 | 33363631 | 72363 | 415 | 61949 | 34 | 10380 | 447 |
| H8 | 85.07 | 52.18 | 83439 | 80112 | 96.01 | 33289891 | 77816 | 415 | 66352 | 114 | 11350 | 246 |
| H9 | 87.13 | 52.33 | 83081 | 80139 | 96.46 | 33172110 | 72731 | 413 | 59891 | 170 | 12670 | 340 |
| H10 | 88.1 | 50.97 | 84428 | 80235 | 95.03 | 34318877 | 79846 | 427 | 72092 | 19 | 7735 | 162 |
| H11 | 85.39 | 52.66 | 84785 | 80041 | 94.4 | 33184085 | 77695 | 414 | 67971 | 31 | 9693 | 279 |
| H12 | 87.38 | 52.46 | 79999 | 78206 | 97.76 | 32985836 | 75672 | 421 | 68059 | 83 | 7530 | 257 |
| H13 | 87.61 | 54.28 | 83039 | 80275 | 96.67 | 33223451 | 75675 | 413 | 65749 | 47 | 9879 | 309 |
| H14 | 85.03 | 55.03 | 86439 | 80121 | 92.69 | 34142471 | 79832 | 426 | 70327 | 6 | 9499 | 219 |
| H15 | 86.4 | 54.19 | 86140 | 80069 | 92.95 | 33117357 | 79440 | 413 | 70008 | 58 | 9374 | 242 |
| H16 | 86.43 | 52.92 | 84676 | 80268 | 94.79 | 33434211 | 72139 | 416 | 60187 | 5 | 11947 | 461 |
| H17 | 87.67 | 53.93 | 85972 | 80213 | 93.3 | 33262011 | 78943 | 414 | 70991 | 6 | 7946 | 249 |
| H18 | 86.77 | 52.46 | 50498 | 44117 | 87.36 | 18442928 | 38829 | 418 | 32529 | 0 | 6300 | 363 |
| H19 | 87.07 | 53.36 | 51912 | 46871 | 90.29 | 19449040 | 42689 | 414 | 36348 | 27 | 6314 | 388 |
| H20 | 84.9 | 52.34 | 55818 | 50355 | 90.21 | 21100565 | 46715 | 419 | 37317 | 21 | 9377 | 300 |
| H21 | 87.25 | 54.14 | 89896 | 84823 | 94.36 | 35224514 | 83452 | 415 | 76169 | 15 | 7268 | 301 |
| H22 | 85.33 | 53.37 | 56514 | 55231 | 97.73 | 22973508 | 54185 | 415 | 47351 | 137 | 6697 | 176 |
| H23 | 84.01 | 50.96 | 75040 | 70443 | 93.87 | 29302580 | 57274 | 415 | 42711 | 54 | 14509 | 329 |
| H24 | 86.79 | 55.44 | 84514 | 80085 | 94.76 | 33089792 | 78738 | 413 | 69121 | 13 | 9604 | 265 |
| xie1 | 85.46 | 49.56 | 65284 | 60619 | 92.85 | 25365592 | 60439 | 418 | 53741 | 0 | 6698 | 236 |
| xie3 | 85.7 | 51.7 | 84884 | 80015 | 94.26 | 34067420 | 78894 | 425 | 68819 | 0 | 10075 | 400 |
| xie6 | 86.79 | 51.21 | 99459 | 95198 | 95.72 | 40490491 | 93827 | 425 | 81896 | 13 | 11918 | 383 |
| xie9.1 | 85.07 | 50.89 | 82636 | 80400 | 97.29 | 34429435 | 78429 | 428 | 67817 | 0 | 10612 | 175 |
| xie9.2 | 79.27 | 48.16 | 57758 | 56289 | 97.46 | 24010989 | 54705 | 426 | 42522 | 489 | 11694 | 184 |
| xie10 | 86.63 | 51.27 | 81369 | 75867 | 93.24 | 32473712 | 74219 | 428 | 65263 | 33 | 8923 | 323 |
| xie13 | 87.68 | 53.86 | 82940 | 80073 | 96.54 | 33283912 | 79926 | 415 | 73816 | 5 | 6105 | 238 |
| xie15.1 | 89.91 | 50.81 | 82728 | 80098 | 96.82 | 34333136 | 79383 | 428 | 75694 | 10 | 3679 | 268 |
| xie15.2 | 84.52 | 52.52 | 68363 | 61804 | 90.41 | 25905271 | 51363 | 419 | 37703 | 5 | 13655 | 568 |
| xie24.1 | 85.89 | 53.21 | 57337 | 54708 | 95.41 | 22770588 | 51860 | 416 | 45490 | 44 | 6326 | 290 |
| xie24.2 | 86.16 | 50.98 | 70159 | 67123 | 95.67 | 28772225 | 66825 | 428 | 62947 | 0 | 3878 | 122 |
| xie26.1 | 84.6 | 51.96 | 59635 | 58429 | 97.98 | 24922175 | 57367 | 426 | 49828 | 1 | 7538 | 255 |
| xie26.2 | 85.46 | 51 | 53649 | 51501 | 96 | 22056233 | 51033 | 428 | 46086 | 7 | 4940 | 336 |
| xie27.1 | 86.1 | 51.29 | 82023 | 80309 | 97.91 | 34445470 | 80219 | 428 | 72811 | 1 | 7407 | 179 |
| xie27.2 | 87.91 | 54.87 | 75316 | 71757 | 95.27 | 29797815 | 71204 | 415 | 65412 | 1 | 5791 | 220 |
| xie31.1 | 88.26 | 50.99 | 83011 | 80137 | 96.54 | 34381233 | 79775 | 429 | 75085 | 2 | 4688 | 208 |
| xie31.2 | 85.22 | 52.32 | 88370 | 81947 | 92.73 | 35190836 | 78921 | 429 | 69585 | 0 | 9336 | 140 |
| xie2* | 85.44 | 51.02 | 81593 | 79547 | 97.49 | 33649892 | 79323 | 423 | 71606 | 0 | 7717 | 217 |
| xie2.1 | 85.8 | 51.12 | 76729 | 72463 | 94.44 | 31064905 | 71789 | 428 | 65511 | 0 | 6278 | 247 |
| xie2.2 | 85.7 | 51.11 | 52298 | 47487 | 90.8 | 20262909 | 45676 | 426 | 38506 | 11 | 7159 | 366 |
| xie4 | 85.07 | 50.83 | 85133 | 80316 | 94.34 | 33494887 | 80085 | 417 | 70622 | 0 | 9463 | 237 |
| xie5.1 | 85.24 | 50.93 | 85473 | 80144 | 93.77 | 34320954 | 78113 | 428 | 67463 | 0 | 10650 | 152 |
| xie5.2 | 85.58 | 51.32 | 85072 | 80091 | 94.14 | 33814114 | 79607 | 422 | 70629 | 0 | 8978 | 252 |
| xie7 | 85.3 | 52.24 | 51401 | 49488 | 96.28 | 20943135 | 48176 | 423 | 42214 | 54 | 5908 | 275 |
| xie8* | 83.57 | 52.7 | 84691 | 79450 | 93.81 | 32936205 | 71723 | 414 | 54213 | 0 | 17510 | 400 |
| xie8.1 | 85.83 | 50.99 | 84031 | 80059 | 95.27 | 34278004 | 77841 | 428 | 66956 | 0 | 10885 | 168 |
| xie8.2 | 85.92 | 51.52 | 85856 | 80086 | 93.28 | 33779619 | 79704 | 421 | 70536 | 0 | 9168 | 243 |
| xie11* | 85.49 | 54.52 | 67841 | 63950 | 94.26 | 26738186 | 45869 | 418 | 29227 | 15 | 16627 | 639 |
| xie11.1 | 84.34 | 50.38 | 83157 | 80070 | 96.29 | 34128199 | 79631 | 426 | 70303 | 0 | 9328 | 229 |
| xie11.2 | 86.19 | 51.01 | 92004 | 83756 | 91.04 | 35706871 | 83635 | 426 | 75375 | 0 | 8260 | 173 |
| xie12.1 | 85.13 | 50.98 | 83567 | 80132 | 95.89 | 34304292 | 77853 | 428 | 66188 | 0 | 11665 | 160 |
| xie12.2 | 82.03 | 51.21 | 53498 | 50808 | 94.97 | 21440917 | 44926 | 421 | 31613 | 479 | 12834 | 342 |
| xie14* | 82.77 | 51.04 | 81264 | 76494 | 94.13 | 32158271 | 73436 | 420 | 59431 | 1134 | 12871 | 383 |
| xie14.1 | 86.18 | 50.95 | 77480 | 70945 | 91.57 | 30358231 | 69143 | 427 | 60193 | 0 | 8950 | 203 |
| xie14.2 | 85.87 | 50.97 | 52185 | 47995 | 91.97 | 20533449 | 46716 | 427 | 40325 | 0 | 6391 | 183 |
| xie16 | 84.98 | 51.41 | 50220 | 42070 | 83.77 | 17971853 | 40056 | 427 | 33427 | 7 | 6622 | 396 |
| xie17* | 86.77 | 50.56 | 86578 | 80224 | 92.66 | 34259266 | 78769 | 427 | 70771 | 42 | 7956 | 318 |
| xie17.1 | 86.08 | 50.95 | 68704 | 67290 | 97.94 | 28815322 | 65650 | 428 | 56963 | 0 | 8687 | 194 |
| xie17.2 | 85.6 | 50.96 | 63243 | 60408 | 95.52 | 25867865 | 58735 | 428 | 49894 | 0 | 8841 | 181 |
| xie18 | 85.48 | 51.58 | 88886 | 83082 | 93.47 | 35424472 | 80892 | 426 | 71257 | 0 | 9635 | 176 |
| xie19 | 86.57 | 53.13 | 60417 | 56043 | 92.76 | 23641507 | 54811 | 421 | 48488 | 46 | 6277 | 320 |
| xie20.1 | 88.81 | 56.69 | 60647 | 59359 | 97.88 | 24334775 | 58601 | 409 | 55306 | 14 | 3281 | 203 |
| xie21.1 | 86.81 | 51.13 | 83587 | 80179 | 95.92 | 34236082 | 78323 | 426 | 72524 | 1 | 5798 | 202 |
| xie21.2 | 85.56 | 52.45 | 74791 | 71947 | 96.2 | 30567899 | 71576 | 424 | 63205 | 4 | 8367 | 194 |
| xie22.1 | 83.41 | 54.51 | 75985 | 71939 | 94.68 | 30709253 | 60089 | 426 | 48337 | 62 | 11690 | 317 |
| xie22.2 | 79.33 | 56.02 | 54975 | 52615 | 95.71 | 22501382 | 40463 | 427 | 28259 | 6 | 12198 | 258 |
| xie23 | 83.16 | 50.83 | 99864 | 91653 | 91.78 | 38824221 | 90489 | 423 | 73323 | 0 | 17166 | 123 |
| xie25 | 85.68 | 52.68 | 63360 | 60921 | 96.15 | 25923651 | 60670 | 425 | 53291 | 1 | 7378 | 222 |
| xie28 | 84.62 | 51.03 | 72460 | 70379 | 97.13 | 29775702 | 70223 | 423 | 61533 | 0 | 8690 | 165 |
| xie29.1 | 85.55 | 53.15 | 69076 | 65105 | 94.25 | 27628437 | 64990 | 424 | 58591 | 0 | 6399 | 161 |
| xie29.2 | 85.97 | 53.47 | 74441 | 70528 | 94.74 | 29940673 | 70357 | 424 | 63866 | 1 | 6490 | 169 |
| xie30.1 | 87.78 | 52.25 | 55075 | 53790 | 97.67 | 22946004 | 53765 | 426 | 50085 | 0 | 3680 | 149 |
| xie30.2 | 87.47 | 55.09 | 84056 | 80820 | 96.15 | 33455845 | 79418 | 413 | 72679 | 0 | 6739 | 197 |
| xie32.1 | 84.82 | 51.57 | 66140 | 64315 | 97.24 | 27134904 | 63794 | 421 | 55823 | 0 | 7971 | 220 |
| xie32.2 | 85.09 | 50.51 | 71292 | 67426 | 94.58 | 27992277 | 66799 | 415 | 57601 | 0 | 9198 | 217 |
| xie33.1 | 85.92 | 52.88 | 71333 | 67004 | 93.93 | 28154534 | 66825 | 420 | 60226 | 0 | 6599 | 164 |
| xie33.2 | 85.48 | 52.59 | 66168 | 61540 | 93.01 | 25947405 | 61416 | 421 | 54436 | 0 | 6980 | 165 |
| xie34 | 86.36 | 51.45 | 62128 | 60015 | 96.6 | 25564522 | 59863 | 425 | 52991 | 1 | 6871 | 181 |
| xie35 | 87.94 | 52.27 | 87743 | 80059 | 91.24 | 33859040 | 79823 | 422 | 71541 | 0 | 8282 | 233 |
| xie36 | 85.66 | 52.73 | 70682 | 68024 | 96.24 | 28837895 | 67862 | 423 | 61039 | 0 | 6823 | 143 |
| xie37 | 85.78 | 53.66 | 84774 | 80320 | 94.75 | 34055114 | 79605 | 423 | 66030 | 2 | 13573 | 451 |
| xie38.1 | 85.77 | 51.44 | 86023 | 80292 | 93.34 | 33621875 | 80188 | 418 | 71957 | 0 | 8231 | 184 |
| xie38.2 | 86.49 | 53.35 | 66591 | 64023 | 96.14 | 26592479 | 63676 | 415 | 58046 | 0 | 5630 | 220 |
| xie39 | 85.5 | 53.43 | 65489 | 63858 | 97.51 | 27107442 | 63584 | 424 | 56615 | 0 | 6969 | 199 |
| xie40.1 | 89.46 | 51.63 | 86500 | 80063 | 92.56 | 34174588 | 78630 | 426 | 71886 | 0 | 6744 | 445 |
| xie40.2 | 83.19 | 51.91 | 86908 | 80169 | 92.25 | 34345933 | 80123 | 428 | 71825 | 0 | 8298 | 131 |
| xie41 | 85.24 | 51.16 | 68443 | 66032 | 96.48 | 28129318 | 65953 | 425 | 58961 | 0 | 6992 | 166 |
| xie42 | 85.41 | 50.89 | 82309 | 80079 | 97.29 | 33638834 | 79821 | 420 | 70237 | 0 | 9584 | 178 |
| xie43.1 | 85.77 | 53.35 | 67218 | 63139 | 93.93 | 26596478 | 63063 | 421 | 57038 | 4 | 6021 | 174 |
| xie43.2 | 85.05 | 52.23 | 75412 | 70411 | 93.37 | 29697997 | 69985 | 421 | 61073 | 0 | 8912 | 190 |
| xie44.1 | 85.24 | 52.34 | 85380 | 79289 | 92.87 | 33565545 | 79006 | 423 | 70271 | 1 | 8734 | 224 |
| xie44.2 | 85.32 | 53.5 | 73127 | 70302 | 96.14 | 29641787 | 70156 | 421 | 62636 | 0 | 7520 | 191 |
| xie45 | 84.99 | 52.14 | 86004 | 81455 | 94.71 | 34417884 | 81282 | 422 | 71924 | 0 | 9358 | 182 |
| xie46 | 85.37 | 51.96 | 54958 | 52023 | 94.66 | 22187880 | 51950 | 426 | 46551 | 0 | 5399 | 156 |
| xie47 | 88.07 | 51.35 | 83006 | 80104 | 96.5 | 34314677 | 79929 | 428 | 73132 | 1 | 6796 | 208 |
| xie48.1 | 85.49 | 52.36 | 107753 | 98149 | 91.09 | 41791650 | 98044 | 425 | 86424 | 0 | 11620 | 174 |
| xie48.2 | 86.27 | 51.11 | 82232 | 80110 | 97.42 | 33199075 | 79975 | 414 | 71905 | 0 | 8070 | 156 |
| xie49 | 87.11 | 52.94 | 88729 | 80171 | 90.35 | 34175052 | 80099 | 426 | 70221 | 0 | 9878 | 123 |
| xie50 | 85.76 | 52.4 | 83882 | 80098 | 95.49 | 33846236 | 79739 | 422 | 69055 | 0 | 10684 | 157 |
| xie51 | 85.25 | 51.79 | 101032 | 94752 | 93.78 | 39832484 | 94549 | 420 | 82342 | 0 | 12207 | 146 |
| xie52 | 82.31 | 50.98 | 82774 | 80046 | 96.7 | 33769188 | 79852 | 421 | 63284 | 0 | 16568 | 154 |

^a^ The five samples marked with an * are blood specimens.

**Table S3. *P* value (Wilcox test) of alpha diversity indices of nasopharyngeal microbiota among groups**

| **Alpha diversity indices** | **Groups** | | |
| --- | --- | --- | --- |
|  | **Healthy vs mild influenza** | **Healthy vs severe influenza** | **Mild vs severe influenza** |
| observed species | 0.0001 | 0.0014 | 0.1467 |
| Chao1 | 0 | 0.0061 | 0.0058 |
| ACE | 0 | 0.0121 | 0.0017 |
| Shannon | 0.6611 | 0.0071 | 0.0195 |
| Simpson | 0.796 | 0.0135 | 0.0212 |
| PD_whole_tree | 0 | 0 | 0.0003 |

**Table S4. The super dominant pathobiontic genus (SDPG) and relative abundance (RA) in nasopharyngeal microbiota**

| **Group and case ID** | **SDPG** | **Relative abundance (RA)** |
| --- | --- | --- |
| Severe group |  |  |
| 1 |  |  |
| 2 | *Acinetobacter* | 96.0% |
| 3 |  |  |
| 4 |  |  |
| 5 | *Lactococcus* | 56.4% |
| 6 |  |  |
| 7 |  |  |
| 8 | *Lactococcus* | 51.4% |
| 9 | *Lactococcus* | 53.7% |
| 10 | *Streptococcus* | 90.2% |
| 11 |  |  |
| 12 | *Lactococcus* | 55.8% |
| 13 | *Corynebacterium* | 68.0% |
| 14 | *Lactococcus* | 60.9% |
| 15 | *Acinetobacter* | 98.2% |
| 16 | *Lactococcus* | 64.1% |
| 17 | *Lactococcus* | 60.2% |
| 18 |  |  |
| 19 |  |  |
| 20 | unidentified*_Corynebacteriaceae* | 97.7% |
| 21 | *Staphylococcus* | 84.3% |
| 22 | *Klebsiella* | 53.8% |
| 23 |  |  |
| 24 | unidentified*_Corynebacteriaceae* | 56.8% |
| 25 | *Streptococcus* | 59.9% |
| 26 |  |  |
| 27 | *Streptococcus* | 96.3% |
| 28 | unidentified*_Prevotellaceae* | 76.7% |
| 29 |  |  |
| 30 | *Acinetobacter* | 66.2% |
| 31 | *Acinetobacter* | 98.8% |
| Mild group |  |  |
| 32 |  |  |
| 33 |  |  |
| 34 |  |  |
| 35 |  |  |
| 36 |  |  |
| 37 |  |  |
| 38 |  |  |
| 39 |  |  |
| 40 | *Pseudomonas* | 72.0% |
| 41 | *Streptococcus* | 51.6% |
| 42 |  |  |
| 43 |  |  |
| 44 |  |  |
| 45 |  |  |
| 46 | *Streptococcus* | 51.3% |
| 47 | *Streptococcus* | 79.2% |
| 48 |  |  |
| 49 | *Streptococcus* | 52.3% |
| 50 |  |  |
| 51 |  |  |
| 52 |  |  |
| Healthy control |  |  |
| H1 |  |  |
| H2 | unidentified*_Corynebacteriaceae* | 66.8% |
| H3 |  |  |
| H4 |  |  |
| H5 |  |  |
| H6 | *Citrobacter* | 57.3% |
| H7 |  |  |
| H8 |  |  |
| H9 |  |  |
| H10 | *Staphylococcus* | 91.5% |
| H11 |  |  |
| H12 | *Staphylococcus* | 56.3% |
| H13 |  |  |
| H14 | *Serratia* | 85.8% |
| H15 | unidentified*_Corynebacteriaceae* | 52.7% |
| H16 |  |  |
| H17 |  |  |
| H18 |  |  |
| H19 |  |  |
| H20 |  |  |
| H21 | unidentified*_Corynebacteriaceae* | 69.3% |
| H22 |  |  |
| H23 |  |  |
| H24 |  |  |

**Table S5. Dominant genus relative abundance (RA) in the nasopharyngeal microbiota and pathogenic bacteria detection**

| **Case ID** | **Dominant Genus in Nasopharyngeal Microbiota (RA%)** | | | **Isolation of Pathogenic Bacteria** | | |
| --- | --- | --- | --- | --- | --- | --- |
|  | **Highest^a^** | **Second** | **Third** | **Blood** | **BLF or ES^b^** | **Sputum** |
| 1 | *Fusobacterium* (30.2%) | *Porphyromonas* (21.6%) | *Streptococcus* (12.1%) |  |  |  |
| 2 | ***Acinetobacter* (96.0%)** | *Klebsiella* (2.0%) | *Pseudomonas* (0.4%) | *Prevotella melaninogenica, Burkholderia cepacia* | ***Acinetobacter baumannii***,  *Klebsiella* *pneumoniae* |  |
|  | *Lactococcus* (54.0%) | *Pseudomonas* (12.2%) | *Acinetobacter* (7.0%) |  |  |  |
| 3 | *Streptococcus* (42.2%) | *Neisseria* (18.0%) | unidentified*_Prevotellaceae* (5.7%) |  |  |  |
| 4 | *Streptococcus* (16.0%) | *Fusobacterium* (15.8%) | *Leptotrichia* (11.8%) |  |  |  |
| 5 | *Lactococcus* (56.4%) | *Pseudomonas* (17.1%) | *Acinetobacter* (10.7%) |  |  |  |
|  | unidentified*_Prevotellaceae* (32.8%) | *Porphyromonas* (18.9%) | *Veillonella* (9.8%) |  |  |  |
| 6 | *Streptococcus* (42.0%) | *Staphylococcus* (16.6%) | unidentified*_Prevotellaceae* (14.8%) | *Streptococcus pyogenes* |  |  |
| 7 | *Staphylococcus* (35.4%) | unidentified*_Corynebacteriaceae* (12.5%) | *Gemella* (10.8%) |  |  |  |
| 8 | *Lactococcus* (51.5%) | *Pseudomonas* (17.5%) | *Acinetobacter* (13.6%) | *K. pneumoniae* |  |  |
|  | unidentified*_Prevotellaceae* (48.0%) | *Leptotrichia* (10.8%) | *Veillonella* (9.2%) |  |  |  |
| 9 | *Lactococcus* (53.7%) | *Pseudomonas* (16.3%) | *Acinetobacter* (13.3%) | *A. baumannii* | *A. baumannii,*  *Aspergillus* |  |
|  | unidentified*_Corynebacteriaceae* (2.1%) | *Psychrobacter* (1.4%) | *Streptococcus* (1.0%) |  |  |  |
| 10 | *Streptococcus* (90.2%) | *Lactococcus* (0.2%) | unidentified_  *Ruminococcaceae* (0.2%) |  |  |  |
| 11 | *Mycoplasma* (36.6%) | *Gemella* (36.0%) | *Peptostreptococcus* (6.7%) |  | *Candida albicans* | *C. albicans* |
|  | *Streptococcus* (53.8%) | *Prevotella* (35.5%) | *Granulicatella* (2.4%) |  |  |  |
| 12 | *Lactococcus* (55.8%) | *Pseudomonas* (16.2%) | *Acinetobacter* (10.1%) |  | *A. baumannii,*  *Pseudomonas aeruginosa* | *A. baumannii,*  *P. aeruginosa,*  *K. pneumoniae* |
|  | *Chryseobacterium* (7.0%) | *Lactobacillus* (7.0%) | *Lactococcus* (6.9%) |  |  |  |
| 13 | unidentified*_Corynebacteriaceae* (68.0%) | *Streptococcus* (14.4%) | *Dolosigranulum* (11.8%) |  | *P. aeruginosa* |  |
| 14 | *Lactococcus* (60.9%) | *Pseudomonas* (14.6%) | *Acinetobacter* (7.9%) |  |  | *A.* *baumannii,*  *K. pneumoniae* |
|  | *Lactococcus* (61.3%) | *Pseudomonas* (12.9%) | *Acinetobacter* (7.7%) |  |  |  |
| 15 | ***Acinetobacter*** (98.2%) | *unidentified_Corynebacteriaceae* (0.2%) | *Wolbachia* (0.1%) | ***A. baumannii*** | ***A. baumannii*** |  |
|  | *Lactobacillus* (11.6%) | *Legionella* (7.5%) | *Streptococcus* (6.8%) |  |  |  |
| 16 | *Lactococcus* (64.1%) | *Pseudomonas* (8.6%) | *Acinetobacter* (4.2%) |  |  | *C. albicans* |
| 17 | *Lactococcus* (60.2%) | *Pseudomonas* (15.4%) | *Acinetobacter* (7.0%) | *Staphylococcus epidermidis* |  |  |
|  | *Lactococcus* (60.5%) | *Pseudomonas* (14.3%) | *Acinetobacter* (7.2%) |  |  |  |
| 18 | unidentified*_Prevotellaceae* (43.4%) | *Veillonella* (20.7%) | *Lactobacillus* (15.6%) |  |  |  |
| 19 | unidentified*_Corynebacteriaceae* (26.1%) | *Streptococcus* (13.7%) | *Rothia* (7.3%) |  |  |  |
| 20 | **unidentified*_Corynebacteriaceae*** (97.7 %) | *Sphingorhabdus* (0.2%) | *Streptococcus* (0.1%) |  | *Candida tropicalis* | ***Corynebacterium striatum****, C. tropicalis* |
| 21 | *Staphylococcus* (84.3%) | *Acinetobacter* (2.6%) | *Cutibacterium* (1.7%) |  |  |  |
|  | *Streptococcus* (42.8%) | *Veillonella* (16.9%) | unidentified*_Prevotellaceae* (11.7%) |  |  |  |
| 22 | *Klebsiella* (53.8%) | *Acinetobacter* (17.1%) | *Veillonella* (16.0%) |  | ***K. pneumoniae*** |  |
|  | ***Klebsiella*** (90.7%) | *Acinetobacter* (1.2%) | *Pantoea* (0.7%) |  |  |  |
| 23 | unidentified*_Prevotellaceae* (47.3%) | *Streptococcus* (15.1%) | *Veillonella* (9.4%) |  |  |  |
| 24 | unidentified*_Corynebacteriaceae* (56.8%) | *Staphylococcus* (11.5%) | *Bacteroides* (4.4%) |  | *K. pneumoniae* | ***A. baumannii,***  *K. pneumoniae* |
|  | ***Acinetobacter*** (96.5%) | unidentified*_Corynebacteriaceae* (0.8%) | *Klebsiella* (0.7%) |  |  |  |
| 25 | *Streptococcus* (59.9%) | *Veillonella* (15.9%) | *Atopobium* (4.6%) |  |  |  |
| 26 | *Neisseria* (17.5%) | *Streptococcus* (17.5%) | *Pseudomonas* (12.9%) |  | ***A. baumannii*** | *K. pneumoniae* |
|  | ***Acinetobacter*** (94.4%) | *Streptococcus* (1.1%) | *Pseudomonas* (0.4%) |  |  |  |
| 27 | *Streptococcus* (96.3%) | unidentified*_Corynebacteriaceae* (1.3%) | unidentified*_*  *Oxyphotobacteria* (0.6%) |  |  | ***C. striatum***,  *C. tropicalis* |
|  | **unidentified*_Corynebacteriaceae*** (70.0%) | *Streptococcus* (19.7%) | unidentified*_Prevotellaceae* (1.7%) |  |  |  |
| 28 | unidentified*_Prevotellaceae* (76.7%) | *Veillonella* (5.7%) | *Streptococcus* (2.5%) |  |  |  |
| 29 | unidentified*_Prevotellaceae* (46.9%) | *Veillonella* (15.3%) | *Actinomyces* (15.0%) |  |  |  |
|  | unidentified*_Prevotellaceae* (46.0%) | *Actinomyces* (18.7%) | *Veillonella* (17.9%) |  |  |  |
| 30 | ***Acinetobacter*** (66.2%) | *Streptococcus* (14.6%) | *Actinomyces* (11.8%) |  | *C. tropicalis* | ***A. baumanni****,*  *C. tropicalis* |
|  | unidentified*_Corynebacteriaceae* (82.2%) | *Staphylococcus* (6.9%) | *Bacteroides* (1.5%) |  |  |  |
| 31 | ***Acinetobacter*** (98.8%) | unidentified*_Lachnospiraceae* (0.1%) | *Neisseria* (0.1%) | *Escherichia coli，*  *Enterococcus faecium* |  | ***A. baumanni*** |
|  | *Acinetobacter* (77.6%) | *Klebsiella* (15.2%) | *Pseudomonas* (5.3%) |  |  |  |
| 32 | *Neisseria* (19.1%) | *Streptococcus* (16.3%) | *Haemophilus* (8.5%) |  |  |  |
|  | *Fusobacterium* (28.6%) | *Leptotrichia* (13.4%) | *Neisseria* (11.3%) |  |  |  |
| 33 | *Leptotrichia* (18.7%) | unidentified*_Prevotellaceae* (18.7%) | *Streptococcus* (17.6%) |  |  |  |
|  | *Veillonella* (28.4%) | unidentified*_Prevotellaceae* (26.8%) | *Leptotrichia* (12.5%) |  |  |  |
| 34 | *Streptococcus* (43.7%) | *Haemophilus* (16.4%) | *Neisseria* (12.2%) |  |  |  |
| 35 | unidentified*_Prevotellaceae* (35.8%) | *Neisseria* (23.6%) | *Leptotrichia* (13.9%) |  |  |  |
| 36 | *Streptococcus* (32.4%) | *Veillonella* (22.8%) | *Rothia* (19.8%) |  |  |  |
| 37 | *Haemophilus* (25.9%) | *Rothia* (24.3%) | *Streptococcus* (22.0%) | *Streptococcus haemolyticus* |  |  |
| 38 | unidentified*_Prevotellaceae* (21.0%) | *Leptotrichia* (14.9%) | *Neisseria* (11.9%) |  |  |  |
|  | unidentified*_Corynebacteriaceae* (20.6%) | *Leptotrichia* (12.8%) | *Rothia* (12.4%) |  |  |  |
| 39 | *Streptococcus* (49.8%) | *Actinomyces* (9.9%) | unidentified*_Prevotellaceae* (9.7%) |  |  |  |
| 40 | ***Pseudomonas*** (72.0%) | *Streptococcus* (10.9%) | *Porphyromonas* (6.4%) |  |  | ***P. aeruginosa*** |
|  | *Actinobacillus* (49.7%) | *Streptococcus* (23.4%) | *Gemella* (20.0%) |  |  |  |
| 41 | *Streptococcus* (51.6%) | unidentified*_Prevotellaceae* (23.1%) | *Leptotrichia* (3.9%) |  |  |  |
| 42 | *Neisseria* (22.1%) | *Leptotrichia* (12.3%) | *Haemophilus* (11.6%) |  |  |  |
| 43 | *Veillonella* (25.1%) | *Rothia* (23.6%) | *Streptococcus* (16.4%) |  |  |  |
|  | *Streptococcus* (24.9%) | *Leptotrichia* (19.2%) | *Aeromonas* (9.7%) |  |  |  |
| 44 | *Streptococcus* (29.7%) | *Neisseria* (14.3%) | *Rothia* (11.3%) |  |  |  |
|  | *Neisseria* (34.1%) | *Rothia* (26.5%) | *Streptococcus* (6.4%) |  |  |  |
| 45 | *Streptococcus* (16.7%) | *Haemophilus* (15.0%) | *Rothia* (12.0%) |  |  |  |
| 46 | *Streptococcus* (51.3%) | *Gemella* (17.8%) | *Rothia* (12.4%) |  |  |  |
| 47 | *Streptococcus* (79.2%) | unidentified*_Prevotellaceae* (8.2%) | *Neisseria* (3.4%) |  |  |  |
| 48 | *Neisseria* (41.0%) | *Streptococcus* (26.1%) | *Actinobacillus* (8.6%) |  |  |  |
|  | *Leptotrichia* (48.8%) | unidentified*_Prevotellaceae* (16.8%) | *Veillonella* (10.3%) |  |  |  |
| 49 | *Streptococcus* (52.3%) | *Veillonella* (19.2%) | *Actinomyces* (10.2%) |  |  |  |
| 50 | *Neisseria* (41.0%) | *Streptococcus* (17.3%) | *Leptotrichia* (16.8%) |  |  |  |
| 51 | unidentified*_Prevotellaceae* (43.8%) | *Veillonella* (15.6%) | *Leptotrichia* (10.0%) |  |  |  |
| 52 | unidentified*_Prevotellaceae* (48.6%) | *Fusobacterium* (9.3%) | *Veillonella* (7.7%) |  |  |  |

^a^ For samples with a SDPG, the genus name and corresponding species name are written in bold font.

^b^ BLF, bronchoalveolar lavage fluid; ES, endotracheal aspirates

**Table S6. information of SNPs in A. baumannii, K. pneumoniae, P. aeruginosa and C. striatum genomes detected in this study.**

|  | ref_base<->  sample_base | ref_codon<->  sample_codon | ref_aa<->  sample_aa | mutate_type | ref_gene_ID | ref_gene_  strand | ref_gene_product |
| --- | --- | --- | --- | --- | --- | --- | --- |
| *A. baumannii* (Reference strain AB030) | | | | | | | |
| NZ_CP009257.1_4181918 | T<->G | ATA<->AGA | I<->R | nonsyn | IX87_21210 | + | alpha/beta fold hydrolase |
| NZ_CP009257.1_1614205 | G<->A | CAG<->TAG | Q<->X | nonsense | IX87_08600 | - | DUF817 family protein |
| NZ_CP009257.1_106214 | G<->A | TTC<->TTT | F<->F | syn | IX87_00665 | - | inorganic phosphate transporter |
| NZ_CP009257.1_1389116 | G<->A | -- | -- | -- | intergenic | -- | -- |
| NZ_CP009257.1_1439041 | A<->G | CTA<->CTG | L<->L | syn | IX87_07560 | + | MgtC/SapB family protein |
| NZ_CP009257.1_1529273 | G<->A | GAA<->AAA | E<->K | nonsyn | IX87_08065 | + | NADP-dependent malic enzyme |
| NZ_CP009257.1_1836711 | T<->C | TAT<->TAC | Y<->Y | syn | IX87_09620 | + | glycosyltransferase family 2 protein |
| NZ_CP009257.1_1987613 | C<->T | CAT<->TAT | H<->Y | nonsyn | IX87_10240 | + | solanesyl diphosphate synthase |
| NZ_CP009257.1_2116735 | A<->G | -- | -- | -- | intergenic | -- | -- |
| NZ_CP009257.1_2220374 | C<->T | TGG<->TGA | W<->X | nonsense | IX87_11440 | - | HAMP domain-containing histidine kinase |
| NZ_CP009257.1_3435899 | C<->T | CGC<->TGC | R<->C | nonsyn | IX87_17440 | + | ribosome-associated protein |
|  |  |  |  |  |  |  |  |
| *K. pneumoniae* (Reference strain HS11286) | | | | | | | |
| NC_016845.1_1504253 | C<->T | TCC<->TCT | S<->S | syn | KPHS_14470 | + | enterobactin synthase subunit E |
| NC_016845.1_2548450 | A<->G | -- | -- | -- | intergenic | -- | -- |
| NC_016845.1_758778 | C<->T | CCG<->CTG | P<->L | nonsyn | KPHS_07190 | + | molybdenum cofactor biosynthesis protein |
| NC_016845.1_1901890 | A<->T | AAA<->ATA | K<->I | nonsyn | KPHS_18340 | + | hypothetical protein |
| NC_016845.1_1905609 | A<->T | -- | -- | -- | intergenic | -- | -- |
| NC_016845.1_2573527 | A<->T | ATT<->TTT | I<->F | nonsyn | KPHS_25500 | + | multiple drug resistance protein MarC |
| NC_016845.1_3331990 | T<->C | ATA<->ACA | I<->T | nonsyn | KPHS_33500 | + | hypothetical protein |
|  |  |  |  |  |  |  |  |
| *P. aeruginosa* ( Reference strain PA01) | | | | | | | |
| NC_002516.2_1593190 | C<->T | CAG<->TAG | Q<->X | nonsense | PA1463 | + | hypothetical protein |
| NC_002516.2_5036891 | A<->C | -- | -- | -- | intergenic | -- | -- |
|  |  |  |  |  |  |  |  |
| *C. striatum* (Reference strain KC-Na-01) | | | | | | | |
| NZ_CP021252.1_231097 | T<->C | AAC<->AGC | N<->S | nonsyn | CBE89_01275 | - | anthranilate phosphoribosyltransferase |
| NZ_CP021252.1_517004 | G<->A | GCA<->ACA | A<->T | nonsyn | CBE89_02520 | + | hypothetical protein |
| NZ_CP021252.1_639094 | T<->C | -- | -- | -- | intergenic | -- | -- |
| NZ_CP021252.1_645534 | A<->G | AAC<->AGC | N<->S | nonsyn | CBE89_03100 | + | phosphoribosylformylglycinamidine cyclo-ligase |
| NZ_CP021252.1_1006176 | T<->C | -- | -- | -- | intergenic | -- | -- |
| NZ_CP021252.1_1276810 | T<->C | AAA<->AGA | K<->R | nonsyn | CBE89_06095 | - | RNA polymerase subunit sigma-70 |
| NZ_CP021252.1_1333074 | G<->T | GCG<->TCG | A<->S | nonsyn | CBE89_06345 | + | 1-deoxy-D-xylulose-5-phosphate synthase |
| NZ_CP021252.1_1337942 | C<->A | CCG<->CCT | P<->P | syn | CBE89_06370 | - | arabinofuranosyl transferase |
| NZ_CP021252.1_1403507 | C<->T | TTC<->TTT | F<->F | syn | CBE89_06720 | + | DNA polymerase I |
| NZ_CP021252.1_1507341 | G<->A | -- | -- | -- | intergenic | -- | -- |
| NZ_CP021252.1_1697336 | C<->A | GCC<->TCC | A<->S | nonsyn | CBE89_08080 | - | electron transfer flavoprotein subunit beta |
| NZ_CP021252.1_1772191 | C<->T | AAC<->AAT | N<->N | syn | CBE89_08410 | + | DUF2269 domain-containing protein |
| NZ_CP021252.1_1904473 | G<->A | GGC<->GGT | G<->G | syn | CBE89_09035 | - | phosphopyruvate hydratase |
| NZ_CP021252.1_1914401 | C<->T | CAG<->CAA | Q<->Q | syn | CBE89_09075 | - | copper oxidase |
| NZ_CP021252.1_2123395 | C<->T | GGC<->GAC | G<->D | nonsyn | CBE89_10060 | - | hypothetical protein |
| NZ_CP021252.1_2165165 | C<->G | CCC<->GCC | P<->A | nonsyn | CBE89_10280 | + | acetolactate synthase |
| NZ_CP021252.1_2295255 | A<->T | CCT<->CCA | P<->P | syn | CBE89_10860 | - | 2-methylcitrate dehydratase |
| NZ_CP021252.1_2295259 | A<->G | TTC<->TCC | F<->S | nonsyn | CBE89_10860 | - | 2-methylcitrate dehydratase |
| NZ_CP021252.1_2435102 | C<->A | GTC<->TTC | V<->F | nonsyn | CBE89_11550 | - | 50S ribosomal protein L6 |
| NZ_CP021252.1_2456565 | C<->A | CCC<->ACC | P<->T | nonsyn | CBE89_11705 | + | hypothetical protein |
| NZ_CP021252.1_2463132 | C<->T | -- | -- | -- | intergenic | -- | -- |
| NZ_CP021252.1_2730053 | G<->T | TCC<->TCA | S<->S | syn | CBE89_13000 | - | hypothetical protein |
| NZ_CP021252.1_1960005 | T<->C | -- | -- | -- | intergenic | -- | -- |
| NZ_CP021252.1_1560803 | C<->T | GAA<->AAA | E<->K | nonsyn | CBE89_07435 | - | transcriptional regulator |
| NZ_CP021252.1_1374438 | C<->T | ACA<->ATA | T<->I | nonsyn | CBE89_06570 | + | carbon starvation protein A |
| NZ_CP021252.1_1125405 | C<->T | GAC<->GAT | D<->D | syn | CBE89_05400 | + | DNA-binding protein |
| NZ_CP021252.1_222657 | G<->A | GCG<->ACG | A<->T | nonsyn | CBE89_01230 | + | oxidoreductase |
